# INSaFLU: an automated open web-based bioinformatics suite “from-reads” for influenza whole-genome-sequencing-based surveillance

**DOI:** 10.1186/s13073-018-0555-0

**Published:** 2018-06-29

**Authors:** Vítor Borges, Miguel Pinheiro, Pedro Pechirra, Raquel Guiomar, João Paulo Gomes

**Affiliations:** 1 Bioinformatics Unit, Department of Infectious Diseases, National Institute of Health, Av. Padre Cruz, 1649-016 Lisbon, Portugal; 2 Institute of Biomedicine—iBiMED, Department of Medical Sciences, University of Aveiro, 3810-193 Aveiro, Portugal; 3 National Reference Laboratory for Influenza and other Respiratory Viruses, Department of Infectious Diseases, National Institute of Health, 1649-016 Lisbon, Portugal

**Keywords:** INSaFLU, Influenza, Next-generation sequencing, Bioinformatics, Variants, Surveillance, Public health

## Abstract

**Background:**

A new era of flu surveillance has already started based on the genetic characterization and exploration of influenza virus evolution at whole-genome scale. Although this has been prioritized by national and international health authorities, the demanded technological transition to whole-genome sequencing (WGS)-based flu surveillance has been particularly delayed by the lack of bioinformatics infrastructures and/or expertise to deal with primary next-generation sequencing (NGS) data.

**Results:**

We developed and implemented INSaFLU (“INSide the FLU”), which is the first influenza-oriented bioinformatics free web-based suite that deals with primary NGS data (reads) towards the automatic generation of the output data that are actually the core first-line “genetic requests” for effective and timely influenza laboratory surveillance (e.g., type and sub-type, gene and whole-genome consensus sequences, variants’ annotation, alignments and phylogenetic trees). By handling NGS data collected from any amplicon-based schema, the implemented pipeline enables any laboratory to perform multi-step software intensive analyses in a user-friendly manner without previous advanced training in bioinformatics. INSaFLU gives access to user-restricted sample databases and projects management, being a transparent and flexible tool specifically designed to automatically update project outputs as more samples are uploaded. Data integration is thus cumulative and scalable, fitting the need for a continuous epidemiological surveillance during the flu epidemics. Multiple outputs are provided in nomenclature-stable and standardized formats that can be explored in situ or through multiple compatible downstream applications for fine-tuned data analysis. This platform additionally flags samples as “putative mixed infections” if the population admixture enrolls influenza viruses with clearly distinct genetic backgrounds, and enriches the traditional “consensus-based” influenza genetic characterization with relevant data on influenza sub-population diversification through a depth analysis of intra-patient minor variants. This dual approach is expected to strengthen our ability not only to detect the emergence of antigenic and drug resistance variants but also to decode alternative pathways of influenza evolution and to unveil intricate routes of transmission.

**Conclusions:**

In summary, INSaFLU supplies public health laboratories and influenza researchers with an open “one size fits all” framework, potentiating the operationalization of a harmonized multi-country WGS-based surveillance for influenza virus.

INSaFLU can be accessed through https://insaflu.insa.pt.

**Electronic supplementary material:**

The online version of this article (10.1186/s13073-018-0555-0) contains supplementary material, which is available to authorized users.

## Background

Influenza virus represents a major public health concern worldwide as it causes annual seasonal epidemics and occasional pandemics leading to high morbidity and mortality in the population [1, 2]. New viral variants emerge constantly due to the never-ending viral genetic and antigenic modification as a consequence of mutation events such as the misincorporation of nucleotides during genome replication or the exchange of genomic segments [3, 4]. The rate of virus evolution is further shaped by the impact of the mutations on the viral fitness as well as by host immunity-related factors or ecological and environmental mechanisms, which ultimately drive the timing and frequency of the emergence of novel epidemic threats [3]. As such, an active molecular-based epidemiological surveillance focused on identifying patterns of viral evolution is a priority in national policies addressing influenza disease prevention, control, and therapeutic measures [3]. To perform the genetic characterization of the virus, public health laboratories have traditionally relied on the Sanger sequencing of hemagglutinin (HA) gene, which only partially covers one of the eight negative-sense single-stranded RNA segments of the virus genome [5]. Moreover, this approach almost exclusively focuses the consensus sequences representing the dominant virus lineage within each infected host at a particular instant, which has limited our knowledge on intra-patient virus population diversity and transmission dynamics [3, 6, 7]. Recently, with the increased availability of next-generation sequencing (NGS) technologies allowing rapid and affordable whole-genome sequencing (WGS), a new era of flu surveillance has started based on genetic analysis of influenza virus at whole-genome scale [8–10]. This transition is expected to reinforce the ability of public health laboratories to (i) monitor genetic profiles of circulating influenza viruses or the emergence of pandemic influenza strains, (ii) detect epitope and antiviral drug resistance mutations, (iii) perform early season risk assessment, (iv) strengthen vaccine effectiveness analysis, and (v) optimize pre-season vaccine strain selection. In this context, there is a growing suite of influenza-specific web platforms that comprehensibly allow, for instance, the annotation of phenotype-associated sequence markers, genotyping or classification of hemagglutinin (HA) clades, the prediction of novel variant proteins, or even the assessment of temporal and geographical virus spread (e.g., Influenza Research Database/Fludb, Nextflu, EpiFLU/GISAID, NCBI Influenza Virus Resource, OpenFluDB) [11–15]. Despite their undeniable usefulness and relevance to the era of NGS-based influenza surveillance, those web-based bioinformatics tools almost exclusively rely on interrogating user-provided sequence or phylogenetic data (downstream steps). In fact, little advance has been achieved to provide public health laboratories with “influenza-specific” bioinformatics tools to deal with primary NGS data (upstream steps), which has been pointed out as the main obstacle for the demanded technological transition for flu surveillance [8]. Many laboratories do not have bioinformatics capabilities and/or staff needed to timely analyze the generated NGS data [8, 16], and, to date, NGS data has been essentially handled through in-house command-line-based pipelines or through wide multi-usage open-source (e.g., Galaxy) or commercial platforms (e.g., Geneious, CLC Genomics Workbench from QIAGEN, Bionumerics from Applied Maths or Ridom SeqSphere+ from Ridom Bioinformatics) [8, 10, 17, 18]. In this context, taking advantage of the recent availability of several multiplex RT-PCR assays for whole-genome amplification of influenza virus [8, 19–24], we built a free bioinformatics web-based suite that deals with primary NGS data (reads) towards the automatic generation of the key genetic output data in a reproducible, transparent, and harmonized manner that fits the disease specificities and short-term goals for (nearly) real-time flu surveillance.

## Implementation

### Overview

**Fig. 1:**
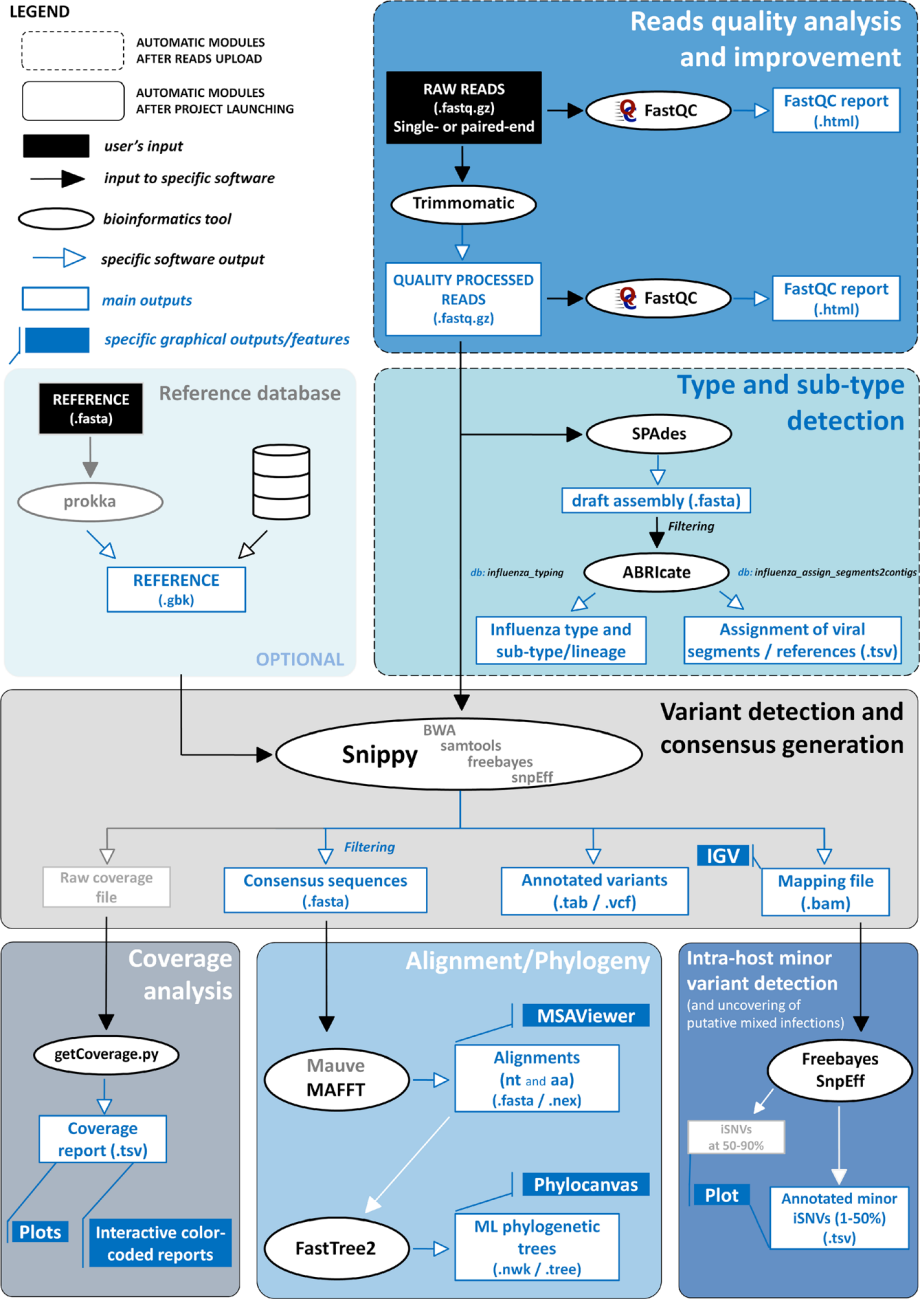
INSaFLU bioinformatics workflow. The diagram (see schematic legend) illustrates all steps of the bioinformatics pipeline developed and implemented in the INSaFLU web platform, enrolling six core modules: (1) read quality analysis and improvement, (2) type and sub-type identification, (3) variant detection and consensus generation, (4) coverage analysis, (5) alignment/phylogeny, and (6) intra-host minor variant detection. Among other features, INSaFLU also determines “putative mixed infections” at two levels: (i) if more than one type, HA or NA subtype or lineage is detected and/or (ii) if the relative proportion of intra-host SNVs at frequency 1–50% (minor iSNVs) and 50–90% satisfies empirically derived criteria (specific alerts are also generated for each case). A detailed description of the INSaFLU outputs is presented in Table 1. Documentation for each module, including software settings and current versions, is provided at the website (https://insaflu.insa.pt)

The bioinformatics pipeline developed and implemented in the INSaFLU web platform currently consists of six core steps: (1) read quality analysis and improvement, (2) type and sub-type identification, (3) variant detection and consensus generation, (4) coverage analysis, (5) alignment/phylogeny, (6) intra-host minor variant detection (and uncovering of putative mixed infections) (Fig. 1). A summary of the INSaFLU current outputs is presented in Table 1. A link [25] to the latest documentation for each module, including software settings and current versions, is provided at the website (https://insaflu.insa.pt) (the documentation at the time this article was published can be found in the Additional file 1; notable changes in INSaFLU platform will be continuously reported in the documentation’s “change log” tab).

**Table 1:** INSaFLU outputs

Module	Output	Format(s)	Description	Tab for visualization/download
Read quality analysis and improvement	FastQC report (raw reads)	.html	FastQC graphical quality reports for raw read files uploaded	Samples/extra info
	FastQC report (quality processed reads)	.html	FastQC graphical quality reports for quality processed reads	Samples/extra info
	Quality processed reads (1P and 2P)	.fastq.gz	Uploaded reads after quality improvement using Trimmomatic	Samples/extra info
Type and sub-type identification	Influenza type and sub-type/lineage	graphical	INSaFLU detects the influenza A and B types, as well as all currently defined influenza A subtypes (18 hemagglutinin subtypes and 11 neuraminidase subtypes) and the two influenza B lineages (Yamagata and Victoria)	Samples/type and subtype (output also included in each project’s “Sample list” table)
	Draft assembly	.fasta	Draft de novo assembly used for type and sub-type/lineage identification. “Influenza-specific” contigs are assigned both to the corresponding viral segments number and to a related reference influenza virus (see next output).	Samples/extra info/type and subtype/lineage identification > “Draft assembly”
	Assignment of viral segments and references	.tsv	Tab-separated file, where each “influenza-specific” NODE (or contig) is assigned both to the corresponding viral segment number (“GENE” column) and to a related reference influenza virus (“ACCESSION” column).	Samples/extra info/type and subtype/lineage identification > “Seg./Ref. to contigs”
Variant detection and consensus generation	Annotated reference file	.gbk	Uploaded reference genome (in .fasta) annotated using Prokka	References/GenBank file
	Mapping file	.bam/graphical	Binary file storing aligned reads to a reference sequence (multi-mapping and unmapped reads are not included); the index is also provided (.bam.bai). “.bam” files can be explored in situ using the Integrative Genomics Viewer (IGV)	Projects/show project results/show sample detail results/mapping file by IGV
	Annotated variants (SNPs and indels) *per* sample	.tab/.vcf	List of annotated variants assumed in the consensus sequences (for each sample)*	Projects/show project results/show sample detail results/mapping file by IGV
	Annotated variants (SNPs and indels) *per* project	.tsv	Compiles all lists of annotated variants assumed in the consensus sequences*	Projects/show project results/project “name” > variants
	Consensus sequences *per* sample (for the pool of loci)	.fasta	A version of the reference sequence with all validated variants replaced. Note: sequences are exclusively generated for locus with 100% of its length covered by ≥ 10-fold)*	Projects/show project results/show sample detail results
Coverage analysis	Coverage report *per* project	.tsv	Compiles the coverage reports for each sample, including the following data: mean depth of coverage *per* locus, % of locus size covered by at least 1-fold and % of locus size covered by at least 10-fold.	Projects/show project results/project “name” > coverage
	Coverage report *per* sample *per* locus (interactive color-coded statistics)	graphical	Green: % of locus size covered by at least 1-fold = 100% and % of locus size covered by at least 10-fold = 100%;	Projects/show project results
			Yellow: % of locus size covered by at least 1-fold = 100% and % of locus size covered by at least 10-fold < 100%;	
			Red: % of locus size covered by at least 1-fold < 100% and % of locus size covered by at least 10-fold < 100%;	
	Coverage report *per* sample *per* locus (plot)	graphical	Plot of the depth of coverage throughout each locus	Projects/show project results
Alignment/phylogeny	Consensus nucleotide alignments *per* locus	.fasta/.nex/graphical	Locus-specific consensus nucleotide alignments. NOTE1: consensus sequences are exclusively generated for locus with 100% of its length covered by ≥ 10-fold). Note 2: The “.fasta” files can be directly uploaded, together with associated metadata (“Sample_list.tsv”), to visualization tools, such as PHYLOViZ.	Projects/show project results/nucleotide alignments by MSAViewer
	Consensus nucleotide alignments—whole genome	.fasta/.nex/graphical	Consensus nucleotide alignments of the “whole genome” sequences (i.e., upon concatenation of all individual locus). Note 1: whole-genome sequences are exclusively generated for samples with all loci with 100% of its length covered by ≥ 10-fold. NOTE2: The “.fasta” files can be directly uploaded, together with associated metadata (“Sample_list.tsv”), to visualization tools, such as PHYLOViZ.	Projects/show project results/nucleotide alignments by MSAViewer
	Consensus amino acid alignments *per* encoded protein	.fasta/.nex/graphical	Consensus amino acid alignments per encoded protein. Note: sequences are exclusively generated for locus with 100% of its length covered by ≥ 10-fold)	Projects/show project results/amino acid alignments by MSAViewer
	Phylogenetic tree *per* locus	.nwk/.tree/graphical	Maximum likelihood phylogenetic tree for each locus-specific nucleotide alignment. NOTE: The “.nwk” and “.tree” phylogenetic trees can be directly uploaded, together with associated metadata (“Sample_list.csv”), to visualization tools, such as Microreact and Phandango, respectively.	Projects/show project results/phylogenetic trees by PhyloCanvas
	Phylogenetic tree*—*whole genome	.nwk/.tree/graphical	Maximum likelihood phylogenetic tree for the alignments of the “whole-genome” sequences (upon concatenation of all individual locus). Note: The “.nwk” and “.tree” phylogenetic trees can be directly uploaded, together with associated metadata (“Sample_list.csv”), to visualization tools, such as Microreact and Phandango, respectively.	Projects/show project results/phylogenetic trees by PhyloCanvas
Intra-host minor variant detection (and uncovering of putative mixed infections)	Annotated minor intra-host single nucleotide variants (iSNVs) *per* project	.tsv	Compiles all lists of detected and annotated minor iSNVs (i.e., SNV displaying intra-sample variation at frequency between 1 and 50% - minor variants).	Projects/show project results/intra-host minor variants annotation and uncovering of mixed infections
	Plots of the proportion of iSNVs at frequencies 1–50% (minor iSNVs) and 50–90%	graphical	Plots the proportion of iSNV at frequency at 1–50% (minor iSNVs) and at frequency 50–90%. You may inspect this plot to uncover infections with influenza viruses presenting clearly distinct genetic backgrounds (so called “mixed infections”). INSaFLU flags samples as “putative mixed infections” if they fulfill the following cumulative criteria: the ratio of the number of iSNVs at frequency 1–50% (minor iSNVs) and 50–90% falls within the range 0.5–2.0 and the sum of the number of these two categories of iSNVs exceeds 20. Alternatively, to account for mixed infections involving extremely different viruses (e.g., A/H3N2 and A/H1N1), the flag is also displayed when he sum of the two categories of iSNVs exceeds 100, regardless of the first criterion.	
Extra	List of samples *per* project	.csv/.tsv	List of samples *per* project (compiles all samples’ metadata and additional INSaFLU outputs). This file can be directly uploaded, together with associated alignment or phylogenetic data, to visualization tools, such as PHYLOViZ, Microreact and Phandango.	Projects/show project results/sample list

*As a conservative approach, consensus sequences are exclusively generated for loci with 100% of its length covered by ≥ 10-fold. Still, validated variants falling within loci not fully covered with ≥ 10-fold, are still included in these lists (these cases are labeled in the column “VARIANTS in INCOMPLETE LOCUS” as YES), so that users can still retrieve valuable and reliable data (e.g., specific epitope and antiviral drug resistance mutations) from samples with borderline coverage

### Read quality analysis and improvement

This module is the first step in almost all WGS bioinformatics analyses and refers to the quality control and improvement of the raw sequencing data. INSaFLU currently accepts single- and paired-end reads (fastq.gz format) generated through widely used NGS technologies, such as Illumina or Ion Torrent. Reads’ quality control in the INSaFLU pipeline is performed by using FastQC software [26], while quality improvement is achieved through Trimmomatic [27]. This tool sequentially (i) performs a trimming sliding window by cutting reads once the average quality within a base window falls below a threshold of quality score, (ii) removes very low-quality bases (or N bases) from both the start and the end of each read if their quality falls below the specified minimum quality required, (iii) excludes reads that fall below a specified length, and (iv) standardize the quality scores by converting them to Phred-33 scores. This first module is automatically run upon reads upload (i.e., no user intervention is needed) and provides the following outputs: (i) FastQC graphical reports (“html” format) of well-established statistics of the reads quality before and after Trimmomatic analysis and (ii) quality processed reads (“fastq.gz” format).

### Type and sub-type identification

In the second step of the pipeline (also automatically run without user involvement), a draft de novo assembly is performed over the quality processed reads using SPAdes [28]. Subsequently, the ABRicate tool [29] is applied to query the draft assemblies against an *in house* database (“influenza_typing”) of a set of type- and sub-type/lineage-specific gene markers that allows the discrimination of the influenza A and B types, all currently defined influenza A subtypes (18 hemagglutinin subtypes and 11 neuraminidase sub-types) and the two influenza B lineages (Yamagata and Victoria). Using this approach, INSaFLU provides the automatic identification of the influenza virus type and sub-type/lineage just after reads upload. Of note, samples are flagged as “putative mixed infections” if more than one type, HA or NA subtype or lineage is detected, and specific alerts are also generated if an incomplete type/subtype is assigned. No incongruence was observed between the in silico determined types or HA subtypes and the result obtained by the traditional “pentaplex” real-time RT-PCR assay applied for influenza diagnosis, typing and sub-typing [30] for the tested the tested 192 A(H3N2) (dataset 1) and 78 A(H1N1pdm09) (dataset 2) viruses. Also notable is that both or either the type and/or sub-type/lineage could be determined for viruses sequenced with very low coverage (mean depth of coverage < 5-fold across the eight amplicons), launching the perspective that this key typing data can be even retrieved from clinical samples with vestigial viruses abundance and/or generating very low PCR yield. The INSaFLU “influenza_typing” database (Additional file 2: Table S1.A) includes (i) representative sequences of the gene encoding the matrix protein (MP or M1 gene) of influenza A and B viruses (to infer the influenza type A or B), (ii) representative sequences of the HA gene of each of the 18 currently defined HA sub-types, (iii) representative sequences of the neuraminidase (NA) gene of each of the 11 currently defined NA sub-types, and (iv) HA representative sequences of the influenza B lineages Yamagata and Victoria. As a proof of concept, all MP, M1, HA, and NA sequences available at Influenza Virus Resource (NCBI) - Influenza Virus Database [31], a total of 184,067 sequences (database accessed in 23–25.10.2017), were screened using the INSaFLU “influenza_typing” tool. The percentage of hits correctly assigned exceeded 99.99% for NA and HA sub-typing and reached 100% for type determination. Of note, this assay detected several types/sub-types mislabeled in the NCBI database (confirmed by BLAST analyses), so these specific mis-discrepancies were not account for specificity estimation purposes. Following the same methodological rationale as described above, draft assemblies are additionally queried against another in house database (“influenza_assign_segments2contigs”) (Additional file 2: Table S1. B) using ABRIcate, enabling the automatic assignment of assembled contigs/nodes to each corresponding viral segment and a closely related reference influenza virus (output is provided as a “.tsv” table). This feature reinforces the application of INSaFLU to (i) analyze viruses for which a closely related whole-genome sequence is not available (e.g., avian influenza) at the INSaFLU or other databases (NICBI, GISAID, etc.), (ii) disclose mixed infections (e.g., by inspecting the output to find if two contigs assigned with same viral segment are flagged with distinct reference influenza viruses), (ii) investigate reassortments (e.g., by inspecting the output to find whether different reference viruses are assigned to different viral segments). Noteworthy, as the database for segments/reference assignment is not as exhaustive as the common influenza sequence repositories (e.g., Influenza Research Database/Fludb, Nextflu, EpiFLU/GISAID), it is prudent that users query those databases or apply other tools (e.g., BEAST, Giraf or BLAST) for specific purposes, such as the detection/confirmation of reassortments or assignment of the closest publicly available sequence of each segment. Yet, the database includes, for instance, representative virus of the circulating 3C.2a and 3C.2a1 genetic sub-groups of seasonal A(H3N2) influenza (as defined by the HA sequence diversity, following ECDC guidelines) as well as representative A(H5N1) viruses from distinct H5 genetic clades, so this INSaFLU feature can promote both the rapid traditional HA genetic sub-group classification and the detection of potential inter- or intra-subtype reassortments during the WGS-based influenza surveillance.

Altogether, upon sample data submission, INSaFLU automatically provides a rapid snapshot of whole-genome backbone of each virus and robustly detects the influenza virus type and sub-type/lineage, which guides the subsequent reference-based downstream module and constitutes an optimal complement to the traditional real-time RT-PCR assays, as it discriminates any HA and NA influenza A sub-types and both influenza B lineages.

### Variant detection and consensus generation

This step of the pipeline consists of mapping the quality processed reads against user-specified reference sequences, followed by SNP/indel calling and annotation, and generation of consensus nucleotide sequences. The current reference database of INSaFLU includes reference sequences of (i) post-pandemic (2009) vaccine-like/reference influenza A(H1N1)pdm2009, A(H3N2) and B viruses (from both Northern and Southern hemispheres) and (ii) representative virus of multiple combinations of HA/NA subtypes (i.e., H1N1, H2N2, H5N1, H7N9, etc.) (check the latest list at the documentation webpage). All reference sequences at INSaFLU are publicly available at NCBI (or made available under permission from the authors). The reference files, both in “.fasta” and “.gbk” (GenBank) format (annotation performed by using Prokka) [32], have been prepared to fit amplicon-based schemas capturing the whole coding sequences (CDS) of the main eight genes of influenza virus (PB2, PB1, PA, HA, NP, NA, M, and NS). Nonetheless, INSaFLU is highly flexible and allows handling NGS data collected from any amplicon-based schema, provided that users fit the reference files to their amplicon design (users just have to generate and upload a multi-fasta file containing reference sequences of the individual amplicons they use with the precise size of the target sequence). Uploaded “.fasta” files are annotated using Prokka upon submission and automatically become available at the user-restricted reference database. In this module, INSaFLU takes advantage of Snippy [33], which is a high flexible multisoftware tool for rapid read mapping (using Burrows-Wheeler Aligner—BWA [34]), SNP- and indel calling (using samtools [35] and freebayes [36]), variant annotation (using SnpEff [37]), and consensus generation (using vcftools [38]). We selected the following criteria for reads mapping and validating SNPs /indels to be annotated, listed and assumed in the consensus sequences: (i) a minimum mapping quality of ≥ 20, (ii) a minimum number of 10 quality processed reads covering the variant position, and (iii) a minimum proportion of 51% of quality processed reads at the variant position differing from the reference. As a conservative approach, for each virus, consensus sequences are exclusively generated for loci with 100% of its length covered by ≥ 10-fold (see below the “Coverage analysis” module for more details), thus avoiding the generation of incomplete sequences that would shrink the nucleotide region available for genetic diversity analyses. Nonetheless, variants that fulfill the above described criteria, but fall within loci not fully covered with ≥ 10-fold, are still included in the list of all variants per sample/project (a specific flag is provided for these cases), so that users can still retrieve valuable and reliable data (e.g., specific epitope and antiviral drug resistance mutations) from samples with borderline coverage. Users can explore all output mapping files (“.bam” format) to view and inspect all reads and variants using the easy-to-use visualization tool Integrative Genomics Viewer [39] available at INSaFLU. These output files are also used in INSaFLU pipeline to more complex downstream analyses (see below the module “Intra-host minor variant analyses”). For each run (see INSaFLU usage section), users must choose the reference sequences (in general, the vaccine-like reference sequences of the season under surveillance) and the pool of samples to be compared (viruses sharing the same type/sub-type as the reference selected, as inferred in the previous module). The option to map reads against same type and sub-type reference sequences of the vaccine reference strains not only potentiates the mapping quality but also has the clear advantage of providing the user with a list of amino acid replacements properly coded to be reported for surveillance. In fact, the amino acid substitutions (including key markers of specific clades/genetic groups) that are reported by National Reference Laboratories to supranational health authorities (e.g., reports to ECDC/WHO via TESSy) are coded against the sequence profile of vaccine-like strains. In summary, this INSaFLU module provides the key data that are actually the core first-line “genetic requests” for effective and timely monitoring of influenza virus evolution on behalf of seasonal influenza laboratory surveillance, i.e., the list of variants (assumed in consensus sequences) and their effect at protein level and also consensus sequences. The latter constitutes the whole basis for the downstream phylogenetic inferences driving the continuous tracking of influenza temporal/geographical spread.

### Coverage analysis

A key standard parameter to take into account when performing NGS is the mean depth of coverage, defined as the mean number of times each base shows up in individual reads (also known as vertical coverage). When handling small amplicon-based NGS data for virus variant detection and consensus generation, it is mandatory to finely inspect the fluctuation of the depth of coverage throughout each amplicon region [6]. Such inspection of the so-called horizontal coverage may not only be highly informative about sequencing-derived artifacts (the coverage plot should typically follow an invert U shape *per* amplicon) but also provides important clues about the degree of relatedness between the genetic background of the “query” virus and the reference sequence chose for mapping. For instance, obtaining sufficient mean depth of coverage for a given amplicon for which its complete length was not covered at 100% may be indicative of miss-mapping due to a high genetic distance between the reference sequence for that locus and the virus under sequencing. These phenomena are typically expected for cases of antigenic shift (reassortment between viral segments from different strains) or intra-segment homologous recombination, or even, for instance, for cases of “mis-subtyping” or “mis-choice” of the reference sequences (e.g., erroneous mapping of A/H1N1pdm09 viruses against a vaccine-like A/H3N2 reference). In this context, we developed the *getCoverage.py* script [40], so that INSaFLU automatically provides the user with a deep analysis of the coverage. Results are provided both *per* sample (graphical outputs) and as batch *per* project (“tsv” format), by yielding the following data: mean depth of coverage *per* locus, % of locus size covered by at least 1-fold, and % of locus size covered by at least 10-fold. The latter statistics was chosen both to fit the minimum depth of coverage for variant calling and to guide the consensus generation (as described above), i.e., the consensus sequences are exclusively provided for amplicons fulfilling the criteria of having 100% of their size covered by at least 10-fold. In addition, INSaFLU interactively yields intuitive color-coded outputs of the coverage statistics as well as depth of coverage plots for each locus *per* sample, enabling users to fine-tune this important parameter towards the uncovering of eventual atypical but highly relevant genetic events, such as reassortment/homologous recombination events.

### Alignment/phylogeny

This module generates harmonized sequence and phylogenetic data that can be directly applied for fine-tuned downstream analysis and visualization platforms, thus promoting the operationalization of a harmonized supranational WGS-based surveillance of influenza virus [8, 41]. First, filtered consensus nucleotide sequences are used as input to progressiveMAUVE [42] and MAFFT [43] for draft and subsequent refined sequence alignment, respectively. INSaFLU provides refined nucleotide sequence alignments (FASTA and NEXUS formats) both at locus level, i.e., for each one of amplicon targets (which are, in general, influenza CDSs), and at “whole-genome” scale (after concatenation of all amplicon targets). Amino acid alignments for annotated proteins are also built using MAFFT [43]. Subsequently, phylogenetic trees (in standard “.nwk” and “.tree” formats) are inferred for each alignment by maximum likelihood under the General Time-Reversible (GTR) model (1000 bootstraps) using double-precision mode of FastTree2 [44]. In order to fulfill the demands of the cumulative data acquisition underlying laboratory surveillance throughout each flu season, for each INSaFLU project, alignments and phylogenetic trees are automatically re-build and updated as more samples are added, making data integration completely flexible and scalable (see “Usage” section). Alignments and phylogenetic trees can be either downloaded for external exploration or explored in situ at INSaFLU website using MSAViewer [45] and PhyloCanvas [46], respectively.

In summary, INSaFLU dynamically builds ready-to-explore scalable gene- and genome-based alignments and phylogenetic trees in standardized nomenclatures and formats that are fully compatible with multiple downstream applications. These include not only other web-based “surveillance-oriented” platforms for influenza genotyping, phenotypic prediction (e.g., Influenza Research Database/Fludb and EpiFLU/GISAID), or phylogeographical/patient data integration (such as, PHYLOViZ, Phandango and Microreact) [47–49], but also several computationally intensive bioinformatics algorithms commonly applied for fine-tuned research of influenza evolutionary dynamics, such as inference of signatures of selection or refined phylogenetics (e.g., the widely used MEGA, DnaSP, BEAST, and RAxML).

### Intra-host minor variant detection (and uncovering of putative mixed infections)

INSaFLU additionally provides the user the possibility to get insight on the influenza intra-patient sub-population dynamics through the scrutiny of minor intra-host single nucleotide variants (iSNVs), i.e., SNV displaying intra-sample frequency below 50%. This is achieved by applying *freebayes* software [36] over mapping files (“.bam” format) with the following criteria: (i) excludes read alignments from analysis if they have a mapping quality of less than 20, (ii) excludes alleles from iSNV analysis if their supporting base quality is less than 20, (iii) requires a minimum of 100-fold depth of coverage to process a site for iSNV analysis, and (iv) requires at least 10 reads supporting an alternate allele within a single individual to evaluate the iSNV frequency. Once fulfilling the above previous criteria, no less than 1% of intra-host frequency of the alternate allele is reported. As such, in a dynamic manner, distinct minimum iSNV frequency cut-offs are assumed depending on the depth of coverage reached at each site, i.e., the identification of iSNV sites at frequencies of 10, 2, and 1% is only allowed if the depth of coverage at a particular site exceeds 100-fold, 500-fold, and 1000-fold, respectively. For each INSaFLU project, results are compiled in a table (“tsv” format) listing all iSNVs (detected for all project’ samples) at frequencies between 1 and 50% (reported frequencies refer to the proportion of reads harboring a nucleotide that is different from the one in the reference). As above, variant annotation (using SnpEff) [37] is also provided. Of note, variants at a frequency above 50%, which correspond to variants included in the consensus sequences, are filtered out from this table since they are systematically listed and annotated upstream in the pipeline (see module “Variant detection and consensus generation”). The table can easily be scrutinized to find sites displaying inter-patient redundancy (i.e., iSNV sites found in more than one individual). These may for instance constitute the ultimate genetic clues for disclosing influenza transmission links [50] or the emergence of antiviral resistance [51, 52]. Similarly to what is outlined in the previous module, this table is automatically re-build and cumulatively updated as more samples are added to each INSaFLU project. In order to additionally enable the detection of infections with influenza viruses presenting clearly distinct genetic backgrounds (so called “mixed infections”), INSaFLU additionally plots the proportion of iSNV at frequency 1–50% (minor iSNVs) and 50–90% detected for each sample (the positional mapping of iSNVs from these two categories within each amplicon can also be explored in the “coverage plots”; see above). A cumulative high proportion of iSNVs at both frequency’ ranges is mostly likely to represent a mixed infection, in a sense that the natural intra-patient influenza diversification is expected to be very low (no more than a few tenths of variants, most of them at frequency < 10%), within the limit of detection of the currently applied NGS techniques [7, 50, 53]. INSaFLU flags samples as “putative mixed infections” based on iSNVs if the following cumulative criteria are fulfilled: the ratio of the number of iSNVs at frequency 1–50% (minor iSNVs) and 50–90% and falls within the range 0.5–2.0 and the sum of the number of these two categories of iSNVs exceeds 20. Alternatively, to account for mixed infections involving extremely different viruses (e.g., A/H3N2 and A/H1N1), the flag is also displayed when the sum of the two categories of iSNVs exceeds 100, regardless of the first criterion. These numerical indicators were empirically inferred upon multiple testing, including the independent NGS run of sample replicates constituting “true” mixed infections (Additional file 3: Figure S1; dataset 1). In order to further consolidate these criteria, an additional proof of concept was carried out by running a bona fide dataset (dataset 3) of artificial mixtures (in triplicate) of A(H3N2) viruses at various proportions previously generated by Shepard and colleagues [17]. INSaFLU was able to detect these same sub-type mixtures at relative frequency of as far as 99:1, as well as yielded match “whole-genome” consensus sequences for all mixtures with the same dominant virus for all triplicates (Additional file 3: Figure S2; dataset 3). Finally, besides this iSNV-based approach, it is also worth noting that samples are also flagged as “putative mixed infections” if more than one type, HA or NA subtype or lineage is detected (see “Type and subtype identification” module).

In summary, through this module, INSaFLU supplies public health laboratories and influenza researchers with relevant data on influenza sub-population diversification within humans that can be systematically integrated in parallel with the “classical” data on “consensus-based” inter-patient virus genetic diversity. Taking into account the recent findings on this subject [50–55], it is expected that this dual approach will strengthen not only our ability to detect the emergence of antigenic and drug resistance variants but also to decode alternative pathways of influenza evolution and to unveil intricate routes of transmission.

### Pre-NGS design and full pipeline testing

The INSaFLU pipeline has been mainly tested with two NGS datasets: 192 samples from A(H3N2) viruses (dataset 1) and 78 samples from A(H1N1) viruses (dataset 2) (see details below). These were generated in an Illumina MiSeq apparatus after influenza whole-genome amplification with a modified wet-lab protocol based on a previously reported RT-PCR assay [19–21]. The adapted pre-NGS protocols, both for influenza A and B viruses, are provided in the INSaFLU’s documentation and can be straightforwardly used for the routine generation of amplicon template for WGS of influenza viruses (irrespective of virus sub-type/lineage). Library preparation was conducted following the Nextera XT DNA Library Prep Reference Guide and WGS runs (96 samples per run) were carried out using MiSeq Illumina flow cells to obtain 2 × 150 paired-end reads (300 cycles). Based on our experience with the described experimental design, success (i.e., 100% of the length of the eight influenza CDS covered by ≥ 10-fold) is largely potentiated if WGS runs are designed to yield > 150,000 (2 × 75,000) reads *per* sample. In fact, above this cut-off, a success of 92% was achieved when comparing with less than 70% obtained for samples with < 150,000 dedicated reads. As a prudent approach, users should design NGS runs to go further this cut-off (e.g., 300,000 reads per sample) in order to better account for issues arising from both the PCR (e.g., fluctuations in the percentage of influenza-specific amplicons across samples and unbalanced relative proportions of the in-sample amplicons) and the NGS run (e.g., low yield and unbalanced demultiplexing of the reads across the samples). INSaFLU modules (relying on robust and widely used software) (Fig. 1) were subjected to specific validation tests to guarantee the generation of accurate outputs, as described above. Still, in order to further attest INSaFLU robustness as a whole, we ran both datasets 1 and 2 with IRMA (v0.6.1; influenza module; default settings) [17], which is the CDC command-line bioinformatics solution for NGS-driven whole-genome assembly and variant detection for RNA viruses, including influenza. Despite using distinct methodological approaches, both platforms start from raw reads towards the generation of the main outputs for influenza surveillance. Comparative analysis of the obtained “whole-genome” consensus sequences using INSaFLU versus IRMA demonstrated similar and robust performance of both pipelines. A detailed description of this assay is presented in Additional file 4: Table S2.

## Results and discussion

Here, we launch INSaFLU, a freely available platform located at the website of the Portuguese National Institute of Health, Instituto Nacional de Saúde (INSA) Doutor Ricardo Jorge, Lisbon, Portugal. It can be openly used upon account creation. This allows data storage/update in a continuous manner, thus facilitating continuous epidemiological surveillance. INSaFLU gives access to private sample and reference databases and projects management. All data is user-restricted, so it will not be viewable by other users. All that is really needed to use INSaFLU is a computer with connection to the Internet. A tutorial providing a complete usage example of data upload, project launching and management, as well as of how to visualize/download graphical and sequence/phylogenetic output data is provided at INSaFLU’s DOCUMENTATION [25] and through a detailed video tutorial available at the INSaFLU homepage. Users can also walkthrough INSaFLU by logging into a “demo” account [56].

### Usage

The web platform architecture is quite intuitive and enrolls the following main tabs: samples, references, and projects.

#### Samples

This menu displays all information for all samples loaded by the user. Required sample-associated data to be uploaded at INSaFLU include the following:

- NGS data: single- or paired-end reads (fastq.gz format) obtained through NGS technologies, such as Illumina or Ion Torrent (reads can be submitted individually or as a batch);
- Sample metadata: a table file can be uploaded for a batch of samples (preferable option) or the sample’s information can be inserted individually at the INSaFLU platform. In order to link the sample data to the uploaded read files, the table file [in comma-separated value (csv) or tab-separated value (tsv)] should contain the columns “sample name”, “fastq1”, “fastq2” (mandatory columns to fulfill; “fastq2” is exceptionally not fulfilled for single-end data) as well these additional variables (that may not be fulfilled), which commonly constitute the typical metadata collected during seasonal influenza surveillance: “data set”, “vaccine status”, “week”, “onset date”, “collection date”, “lab reception date”, “latitude”, “longitude”. However, users may include any other columns with metadata variables to be associated with samples. An example table file is provided at the website. The option to upload tables enriched with multiple metadata variables has the clear advantage of allowing their subsequent direct upload (along to the standardized and multi-format outputs of INSaFLU: alignments/trees) to downstream platforms for phylogenetic data visualization and/or phylogeographical analysis, such as PHYLOViZ [45], which accepts sample metadata (tab-separated format) plus alignments (FASTA format), Phandango [46], which runs sample metadata (csv-separated format) and a phylogenetic tree (“.tree” format) or Microreact [47], which takes sample metadata (in csv-separated format) plus a phylogenetic tree (“.nwk” format).

Upon submission, INSaFLU automatically updates samples’ information with read’s quality and typing data, as well as provides a rapid snapshot of whole-genome backbone of each virus by assigning influenza segments and references to a draft assembly.

#### References

This menu displays all information for all reference sequences available at user’s confidential account. INSaFLU provides a default reference database including publicly (NCBI) available (or made available under permission from the authors) sequences from several post-pandemic (2009) vaccine-like/reference virus and representative virus of multiple combinations of HA/NA subtypes. The database includes whole-genome sequences (FASTA and GenBank formats) that are ready to be used for reference-based mapping (see next section). Nonetheless, users are allowed to upload additional reference files to a user-restricted reference database (uploaded “.fasta” files are automatically annotated upon submission).

#### Projects

This menu allows the creation of scalable projects relying on the selection of (i) a reference file from the reference database that fit their amplicon design (i.e., a multi-fasta file containing reference sequences of the individual amplicons they use with the precise size of the target sequence) and (ii) the batch of samples to be included in the project. Since the projects are scalable, users are encouraged to create “umbrella” projects, such as projects enrolling the mapping of all same sub-type virus against the vaccine-like reference virus for a given flu season. Outputs of the project are organized by dynamic “expand-and-collapse” panels that allow a user-friendly visualization/download of all graphical and sequence output data.

### Benefits

INSaFLU is, to the best of our knowledge, the first influenza-oriented bioinformatics open web-based suite that deals with primary NGS data (reads) towards the automatic generation of the output data that are actually needed for the first-line influenza surveillance (type and sub-type, gene and whole-genome sequences, alignments and phylogenetic trees). The main advantages offered by INSaFLU are the following:

(i) It allows handling NGS data collected from any amplicon-based schema;
(ii) It enables laboratories to perform advanced, multi-step software intensive analyses in a user-friendly manner without previous advanced training in bioinformatics;
(iii) It is freely available tool that and can be used upon account creation giving access to user-restricted sample and reference databases and projects management;
(iv) It is located at the website of a National Institute of Health, which ensures confidentiality and ethics;
(v) It is a flexible tool specifically designed to integrate output data in a cumulative manner, thus fitting the analytical dynamics underlying a continuous epidemiological surveillance during the flu epidemics;
(vi) Outputs are provided in nomenclature-stable and standardized format and can be explored in situ or through multiple compatible downstream applications for fine-tuned data analysis.

### Future directions

INSaFLU was designed to overcome a major caveat in field which is the lack of tools for automate manipulation of raw NGS data for flu whole-genome-based surveillance. Still, this platform is under active development in order to have additional features, such as modules to automatically detect virus reassortment, and perform temporal and geographical data integration and visualization.

## Conclusions

INSaFLU provides an open “one size fits all” framework that guarantees that the application of WGS-based bioinformatics for flu surveillance can be easily accessed by any laboratory around the world with a common computer with access to Internet. It will certainly strengthen the detection of genetic changes in circulating influenza viruses, the detection of potential pandemic influenza strains, the early season risk assessment and vaccine effectiveness analysis, the detection of genetic markers associated with antiviral resistance, and pre-season vaccine strain selection. Ultimately, INSaFLU has the potential to facilitate collaborative initiatives among cross-sectorial stake-holders enrolled in the flu surveillance, with benefits for public health.

## Additional files

Additional file 1: INSaFLU’s Documentation. PDF file containing the INSaFLU documentation (hosted in “Read the Docs”; https://readthedocs.org/) at the time this article was published; documentation will be kept updated at http://insaflu.readthedocs.io/en/latest/, where notable changes in INSaFLU platform will be continuously reported in the “Change log” tab. (PDF 9136 kb)

Additional file 2: **Table S1.** A. INSaFLU genetic markers for type and subtype/lineage identification (“influenza_typing” database). B. INSaFLU genetic markers for the assignment of segments (and references) to draft contigs (“influenza_assign_segments2contigs” database). GISAID acknowledgement tables are included in these tables. (XLSX 45 kb)

Additional file 3: **Figure S1.** INSaFLU graphical output plotting the number of iSNVs at frequencies 1–50% (minor iSNVs) and 50–90% obtained for dataset 1. **Figure S2.** INSaFLU testing with artificial mixtures of A(H3N2) viruses. A. INSaFLU graphical output plotting the number of iSNVs at frequencies 1–50% (minor iSNVs) and 50–90%. B. Phylogenetic tree based on “whole-genome” consensus sequences obtained for dataset 3. (PDF 977 kb)

Additional file 4: **Table S2.** A. Matrix of pairwise nucleotide differences between whole-genome consensus sequences obtained using INSaFLU versus IRMA for 137 A(H3N2) viruses from dataset 1. B. Matrix of pairwise nucleotide differences between whole-genome consensus sequences obtained using INSaFLU versus IRMA for 39 A(H1N1pdm09) viruses from dataset 2. (XLSX 100 kb)

## Abbreviations

CDS: Coding sequence
csv: Comma-separated value
ECDC: European Centre for Disease Prevention and Control
HA: Hemagglutinin
INSaFLU: INSide the FLU
iSNVs: Intra-host SNV
NA: Neuraminidase
NGS: Next-generation sequencing
RT-PCR: Reverse transcription polymerase chain reaction
SNP: Single nucleotide polymorphism
SNV: Single nucleotide variant
tsv: Tab-separated value
WGS: Whole-genome sequencing
WHO: World Health Organization
