# Supplementary material for: INSaFLU: an automated open web-based bioinformatics suite “from-reads” for influenza whole-genome-sequencing-based surveillance
Source: Genome Med. 2018 Jun 29;10:46. doi: 10.1186/s13073-018-0555-0 (PMC6027769; doi:10.1186/s13073-018-0555-0)
Supplement: Supplementary file 1 — INSaFLU’s Documentation. PDF file containing the INSaFLU documentation (hosted in “Read the Docs”; https://readthedocs.org/) at the time this article was published; documentation will be kept updated at http://insaflu.readthedocs.io/en/latest/, where notable changes in INSaFLU platform will be continuously reported in the “Change log” tab. (PDF 9136 kb) [file 13073_2018_555_MOESM1_ESM.pdf]

Additional file 1

INSaFLU's Documentation. PDF file containing the INSaFLU documentation at the time this article was published; documentation will be kept updated at http://insaflu.readthedocs.io/en/latest/, where notable changes in INSaFLU platform will be continuously reported in the "Change log" tab.

This document was downloaded using "Read the Docs" functionalities (https://readthedocs.org/), where **INSaFLU's Documentation is hosted.**

INSaFLU Documentation

Release 0

Vitor Borges

Jun 05, 2018

Contents

1 Highlights **3**

2 Contact **5**

3 Contents: **7**

3.1 Guide for pre-NGS steps . . . 7
3.2 Uploading data . . . 8
3.3 Project creation and scaling-up . . . 19
3.4 Data analysis . . . 22
3.5 Output Visualization and Download . . . 30
3.6 Change log . . . 43

INSaFLU ("INSide the FLU") is an influenza-oriented bioinformatics free web-based suite that deals with primary data (reads) towards the automatic generation of the output data that are actually the core first-line "genetic requests" for effective and timely influenza laboratory surveillance.

https://insaflu.insa.pt

CHAPTER 1

Highlights

- open to all, free of charge, user-restricted accounts
- applicable to NGS data collected from any amplicon-based schema
- allows advanced, multi-step software intensive analyses in a user-friendly manner without previous training in bioinformatics
- automatic identification of influenza type and subtype/lineage, detection of putative mixed infections and intra-host minor variants
- allows integrating data in a cumulative manner, thus fitting the analytical dynamics underlying the continuous epidemiological surveillance during flu epidemics
- outputs are provided in nomenclature-stable and standardized formats and can be explored in situ or through multiple compatible downstream applications for fine-tune data analysis and visualization

CHAPTER 2

Contact

If you have any questions, comments or suggestions, please contact us: <vitor.borges@insa.min-saude.pt> or <j.paulo.gomes@insa.min-saude.pt>

CHAPTER 3

Contents:

3.1 Guide for pre-NGS steps

3.1.1 Suggested pre-NGS wet-lab protocol

INSaFLU is highly flexible and **allows handling NGS data collected from any amplicon-based schema**, provided that users fit the reference files to their amplicon design (users just have to generate and upload a multi-fasta file containing reference sequences of the individual amplicons they use with the precise size of the target sequence).

The default reference database of INSaFLU currently includes reference sequences of:

1. post-pandemic (2009) vaccine/reference influenza A(H1N1)pdm2009, A(H3N2) and B viruses (from both Northern and Southern hemispheres);
2. representative virus of multiple combinations of HA/NA subtypes (i.e., H1N1, H2N2, H5N1, H7N9, etc)

All reference sequences at INSaFLU are publicly available at NCBI (or are made available under permission of the authors). Download the current list here: `INSaFLU_current_REFERENCE_DATABASE_11_05_2018.xlsx`)

The reference files have been prepared to fit amplicon-based schemas capturing the whole CDS of the main eight genes of influenza virus (PB2, PB1, PA, HA, NP, NA, M and NS).

INSaFLU pipeline has been tested with NGS data collected after applying the wet-lab pre-NGS protocol for influenza whole genome amplification adapted from a RT-PCR assay described by Zhou and colleagues (Zhou et al, 2009, for Influenza A; and Zhou et al, 2014, for Influenza B; Zhou and Wentworth, 2012). This protocol can be applied to simultaneously amplify the eight genomic RNA segments, irrespective of influenza virus subtype or lineage.

You can download the suggested protocol here: `Suggested_RT_PCR_assay_for_influenza_WGS.pdf`

3.1.2 How to design a NGS run?

We suggest you ask your NGS service provider to perform runs in order to yield a final output of about 300000 (2 x 150000) reads per sample.

This will account for issues arising from both the PCR reactions (e.g., fluctuations in the percentage of influenza-specific amplicons across samples and unbalanced relative proportions of the in-sample amplicons) and the NGS run (e.g., low yield and unbalanced demultiplexing of the reads across the samples).

This approach will allow you to end-up with more than 150000 (2 x 75000) reads per sample. This cut-off yielded a success (i.e., sample with 100% of the length of the 8 influenza CDS covered by 10-fold) of 92% on our pilot study using 2 x 150 paired-end reads (300 cycles).

Note: Examples of Illumina MiSeq runs that fit this suggestion are:

1. run 96 samples using Illumina V2 Standard flow cells (30 M reads total; 300 cycles);
2. run 24 samples using Illumina Micro flow cells (4 M reads total; 300 cycles).

References:

- Zhou B, Donnelly ME, Scholes DT, St George K, Hatta M, Kawaoka Y, Wentworth DE. 2009. Single-reaction genomic amplification accelerates sequencing and vaccine production for classical and Swine origin human influenza a viruses. J Virol, 83:10309-13.
- Zhou B, Lin X, Wang W, Halpin RA, Bera J, Stockwell TB, Barr IG, Wentworth DE. 2014. Universal influenza B virus genomic amplification facilitates sequencing, diagnostics, and reverse genetics. J Clin Microbiol, 52:1330-1337.
- Zhou B, Wentworth DE. 2012. Influenza A virus molecular virology techniques. Methods Mol Biol, 865:175-92.

3.2 Uploading data

INSaFLU needs:

- **NGS data** (fastq reads) (**mandatory**)
- **Sample metadata** (to link each sample to the respective NGS data) (**mandatory**)
- **Reference data** (additional user-restricted reference sequences) (*optional*)

3.2.1 Uploading Sample metadata and NGS data

Samples's metadata and respective **NGS data** (single-end or paired-end reads in fastq.gz format obtained through widely used technologies, such as Illumina or Ion Torrent) can be uploaded to INSaFLU as a **batch (option 1)** or **individually (option 2)**:

Option 1 (Batch)

A. Go to *Samples* menu and choose *Add Samples from csv / tsv file*.

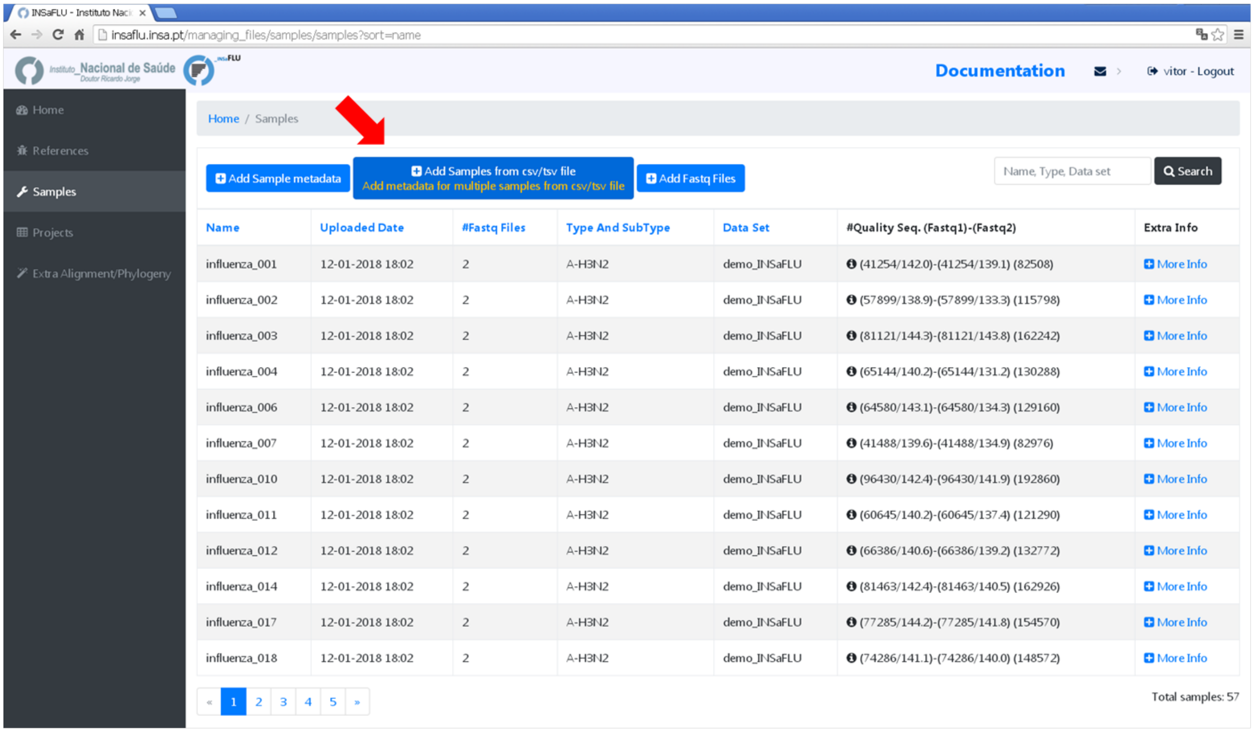

Check your list of "Samples metadata" tables and **Load a new file**:

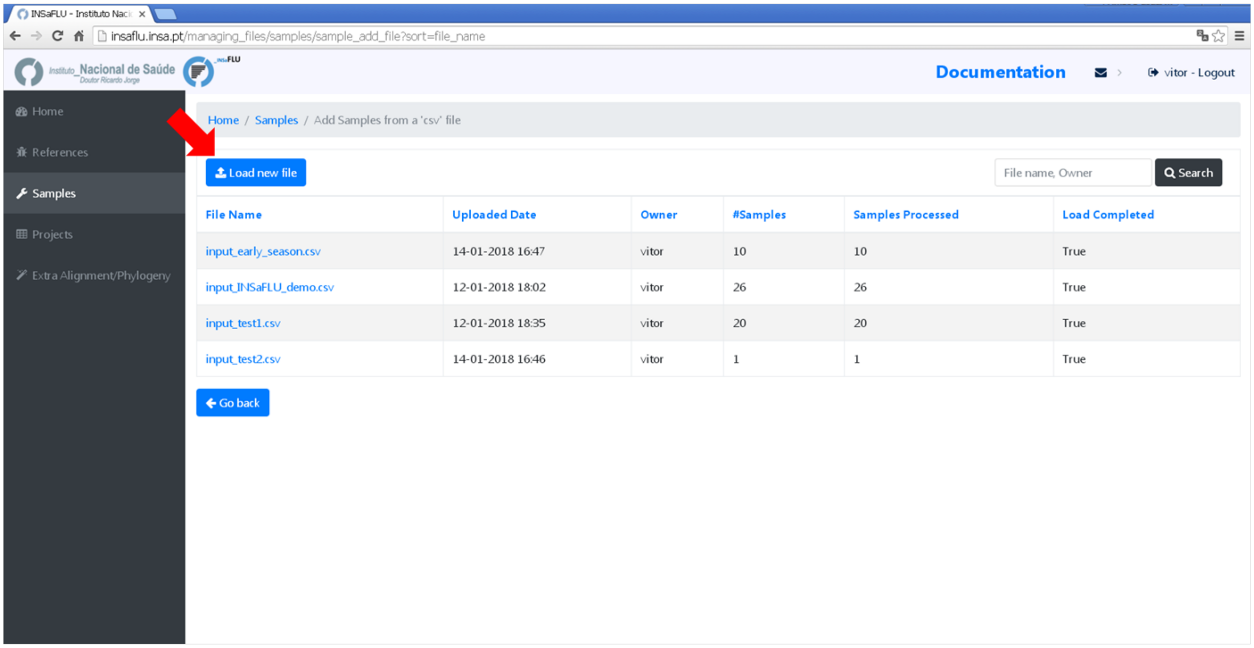

Examples of template table files are provided in this menu.

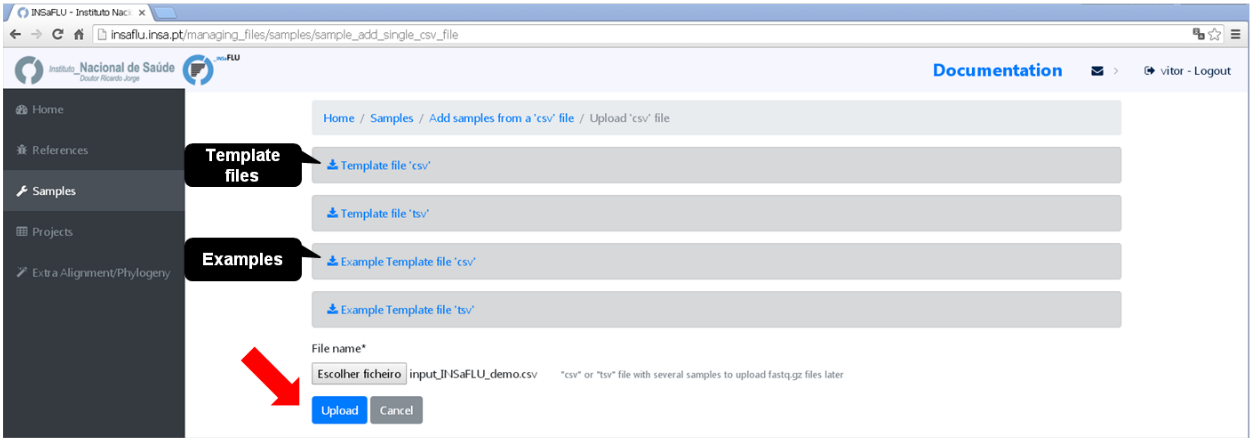

Important: Sample metadata should be a comma-separated value (.csv) or tab-separated value (.tsv or .txt) table containing the columns **"sample name"**, **"fastq1"** and **"fastq2"** (**mandatory columns to fulfill**; NOTE: fastq2 is exceptionally not fulfilled only for single-end data) as well these additional variables (that may not be fulfilled): **"data set"**, **"vaccine status"**, **"week"**, **"onset date"**, **"collection date"**, **"lab reception date"**, **"latitude"**, **"longitude"**.

Users are encouraged to include any other columns with metadata variables to be associated with samples (see advantages below).

Important: Samples names in your account must be unique and only numbers, letters and underscores are allowed.

Fastq.gz file names must be complete (including extension) and match the ones from the files that you are going to upload later.

Advantages of uploading enriched Sample metadata tables

The template file contains the following additional variables, which commonly constitute the minimal metadata collected during seasonal influenza surveillance: "data set", "vaccine status", "week", "onset date", "collection date", "lab reception date", "latitude" and "longitude". (The "data set" column will help you to organize samples by specific groups, e.g., "early_season", "peak", etc)

The option to upload tables enriched with multiple metadata variables has the clear advantage of allowing the subsequent upload of the samples list of each project (output: "Sample_list.csv" or "Sample_list.tsv") along to the standardized and multi-format outputs of INSaFLU projects (alignments/trees) to downstream platforms for phylogenetic data visualization and/or phylogeographical analysis, such as:

- PHYLOViZ (http://www.phyloviz.net/), which accepts sample metadata (tab-separated format) plus alignments (FASTA format), among other formats;
- Phandango (https://jameshadfield.github.io/phandango/#/), which runs sample metadata (csv-separated format) plus a phylogenetic tree (".tree" format);
- Microreact (https://microreact.org/), which takes sample metadata (in csv-separated format) plus a phylogenetic tree (".nwk" format).

Note: To take full advantage of particular platforms, such Microreact, which provides a framework where associated data (geographical, temporal, phenotypic or epidemiological) can be easily explored together with phylogenetic data, users may need to specify additional columns in the table (e.g. specifying "year", "month" and "day" columns, along with "latitude" and "longitude" allows Microreact to automatically integrate temporal and geolocation data;). Also, for colour/shape definitions, you will need to create extra columns (see Microreact instructions here: https://microreact.org/instructions).

It is worthnoting that, for Microreact, depending on the tree (whole-genome or locus-specific trees) that you use (which can include a distinct set of viruses), you will additionally need to match the number of identifiers within the id column to the number of viruses in each tree.

B. Go to *Samples* menu and choose *Add Fastq Files*

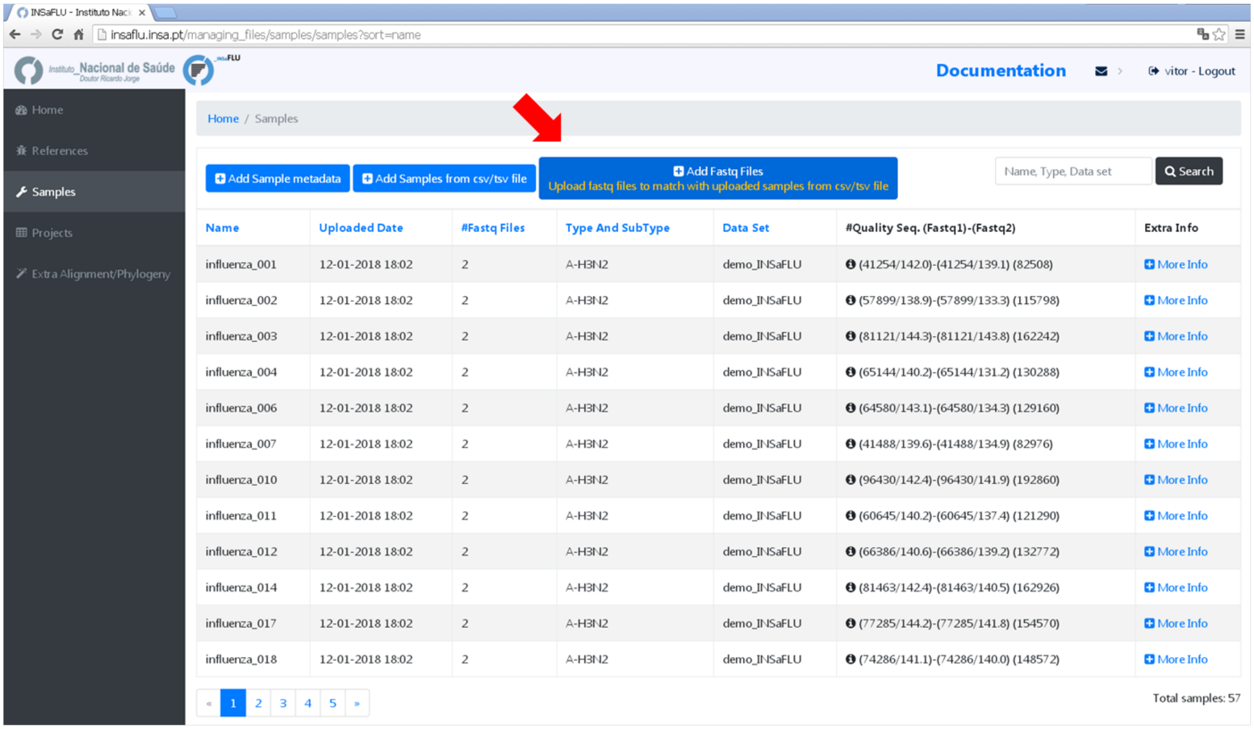

Navigate through your list of fastq.gz files and **simultaneously upload multiple fastq.gz files** (300MB maximum per file; files between 50-300 MB are downsized to ~50 MB by randomly sampling reads), which are automatically linked to the corresponding samples.

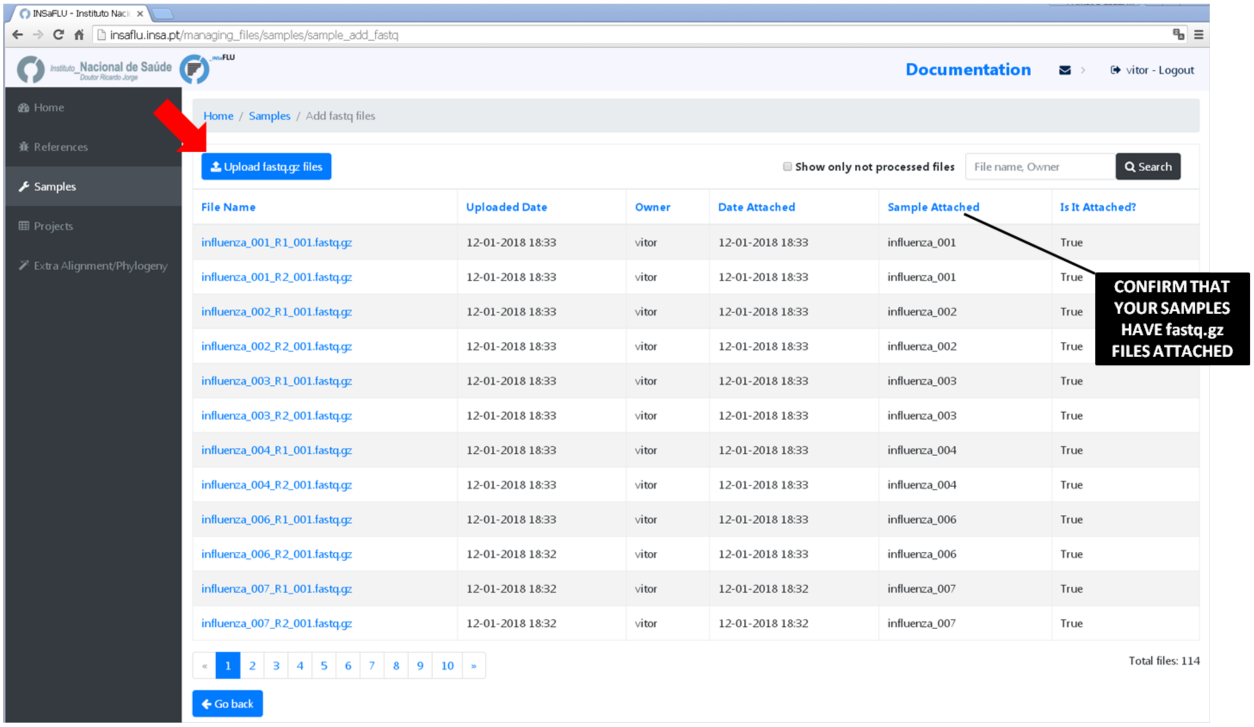

Just DRAG & DROP your fastq.gz files

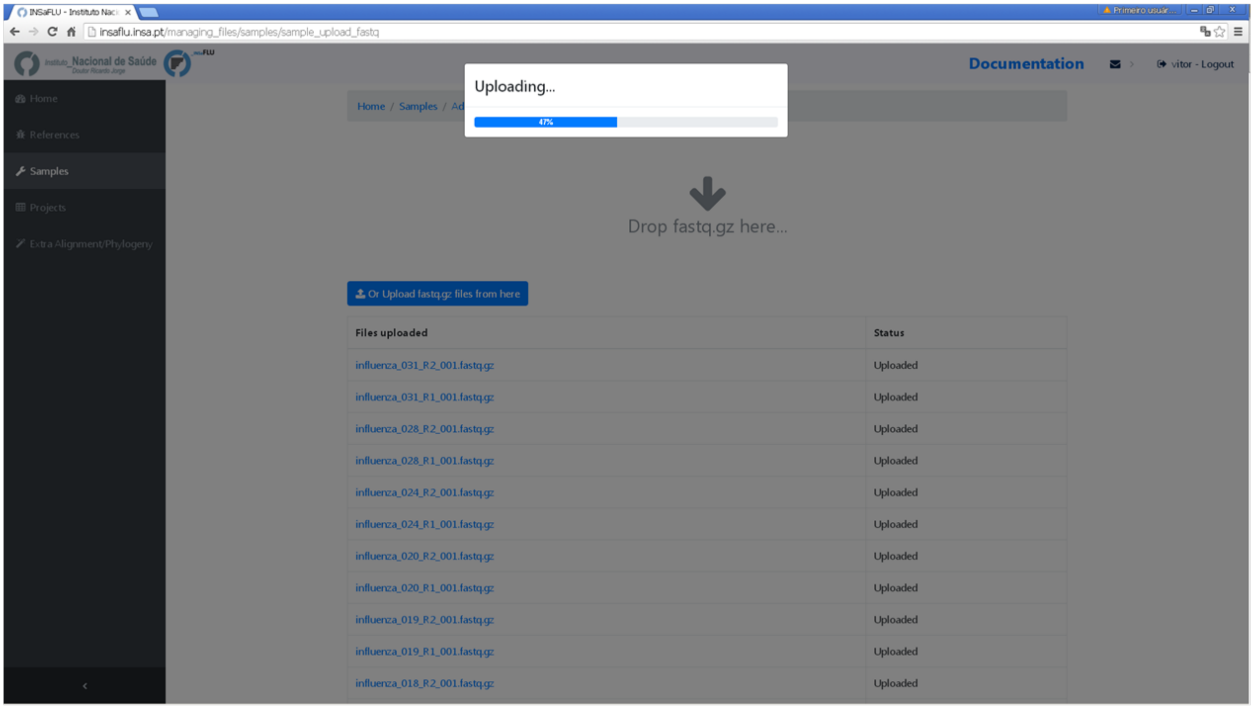

Option 2 (Individual)

A. Go to *Samples* menu and choose *Add Sample metadata*

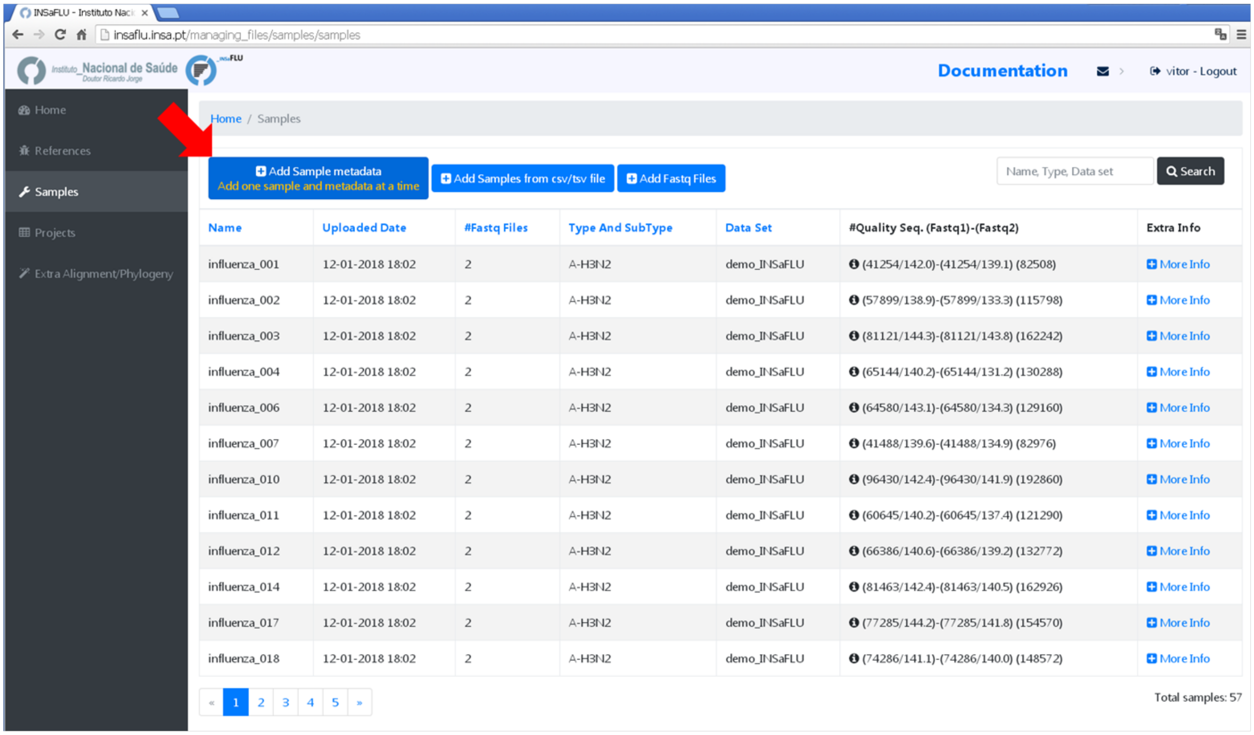

Here you can upload each sample at the time (including associated metadata and NGS data).

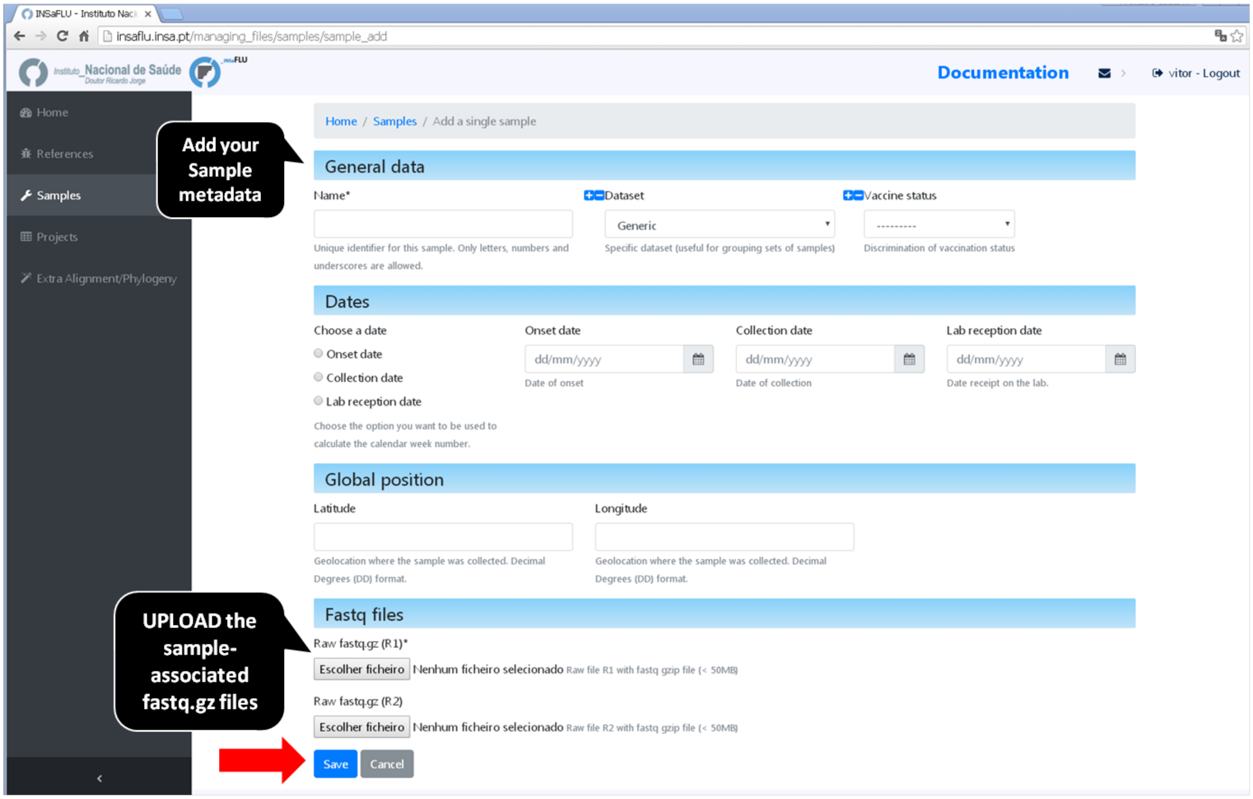

3.2.2 Uploading Reference data

INSaFLU needs reference sequence files to be used for reference-based mapping.

- In *References* menu, **INSaFLU provides a set of ready-to-use reference sequences**, all publicly available at NCBI (or made available under permission of authors), currently including:

  1. post-pandemic (2009) vaccine/reference influenza A(H1N1)pdm2009, A(H3N2) and B viruses (from both Northern and Southern hemispheres);
  2. representative virus of multiple combinations of HA/NA subtypes (i.e., H1N1, H2N2, H5N1, H7N9, etc)

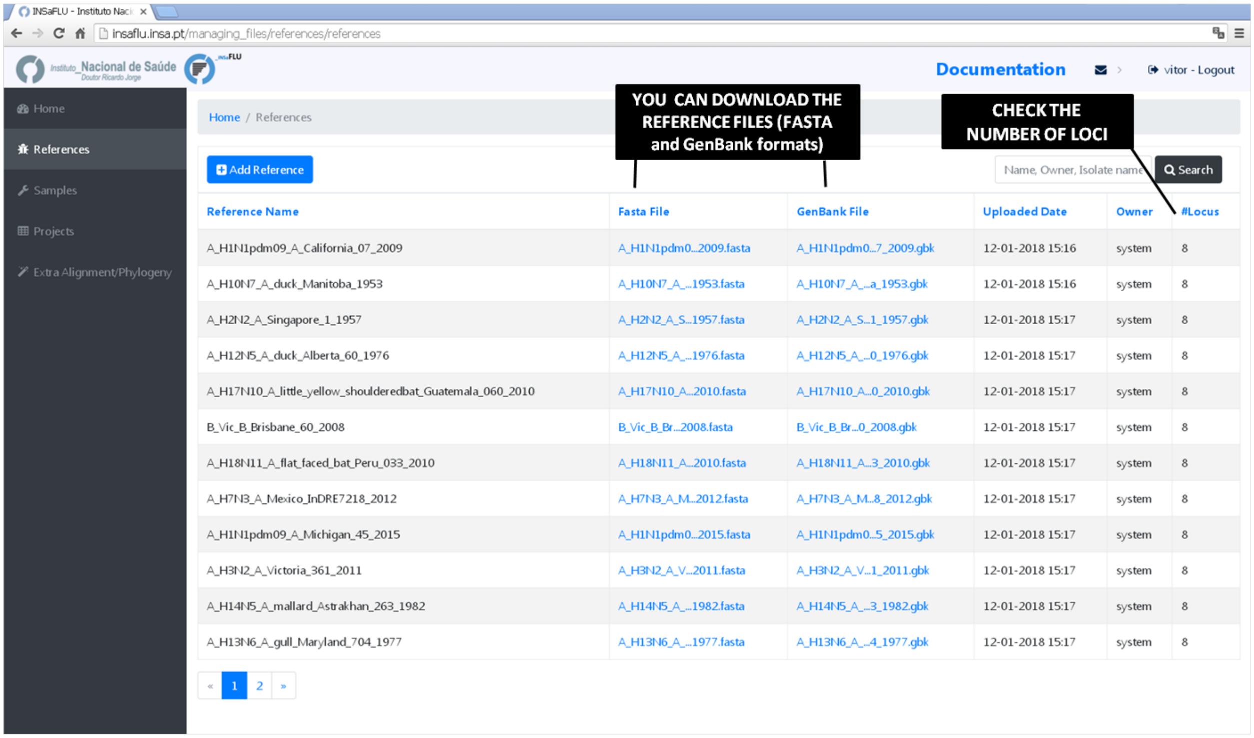

The current list of reference sequences, including loci size and NCBI accession numbers is provided here:

`INSaFLU_current_REFERENCE_DATABASE_11_05_2018.xlsx`

The default reference files (FASTA and GenBank formats) have been prepared to fit amplicon-based schemas capturing the whole CDS of the main eight genes of influenza virus (PB2, PB1, PA, HA, NP, NA, M and NS), such as the wet-lab pre-NGS protocol for influenza whole genome amplification adapted from a RT-PCR assay described by Zhou and colleagues (Zhou et al, 2009, for Influenza A; and Zhou et al, 2014, for Influenza B; Zhou and Wentworth, 2012).

You can download the suggested protocol here: `Suggested_RT_PCR_assay_for_influenza_WGS.pdf`

Important: NO FURTHER ACTIONS ARE NEEDED if you are using the suggested wet-lab pre-NGS protocol and you want to compare your sequences against a reference available at INSaFLU database.

However, you may need to UPLOAD additional reference files to the user-restricted reference database. For instance, you may need to upload the A/H3N2 vaccine reference sequence for the season 2017/2018 (A/Hong Kong/4801/2014 virus), which is not freely available.

To upload additional references (FASTA format; maximum 50000 bp per file): GO TO *References* MENU and CHOOSE **Add Reference**

See below a guide to generate additional reference sequences

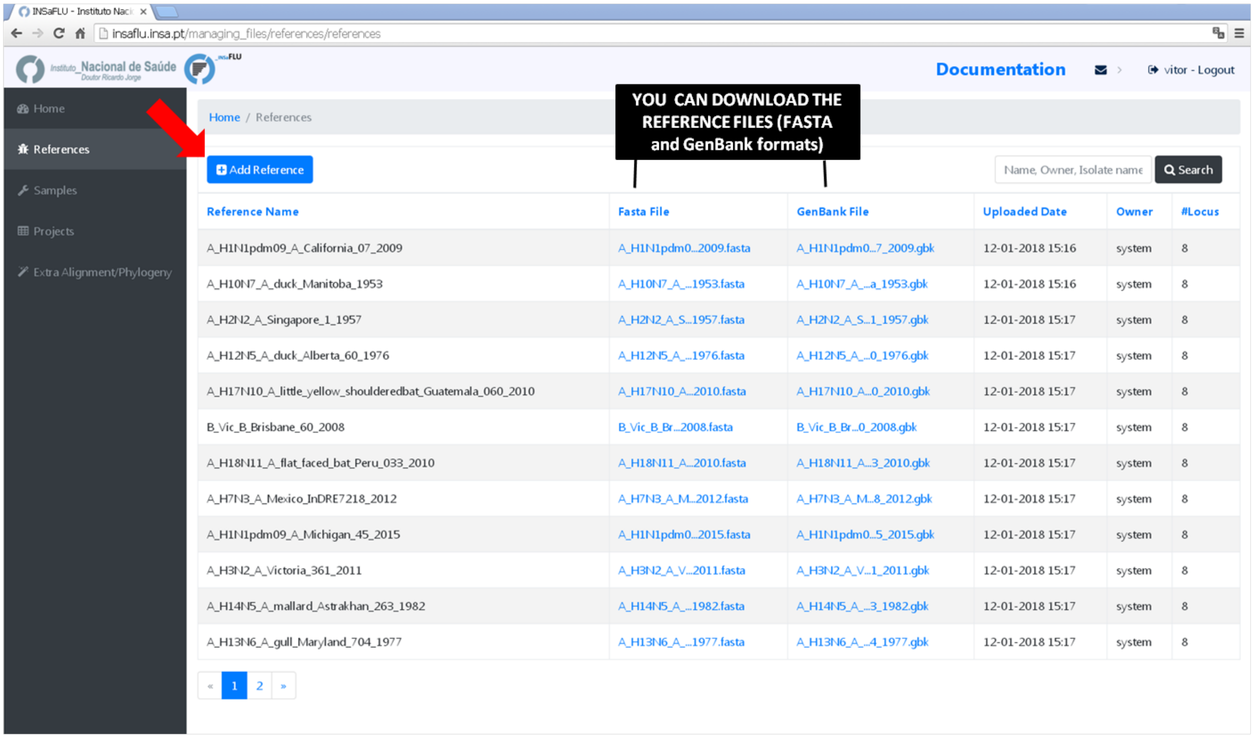

Note: You can upload:

1. multi-FASTA files containing the set of reference sequences that constitute the influenza "whole-genome" sequence of a particular virus (e.g, the combination of the traditional 8 amplicons targeting the 8 eight influenza RNA segments). Each individual sequence must have the precise size of each "intra-amplicon" target sequence that you capture by each one of the RT-PCR amplicons. INSaFLU automatically annotates uploaded multi-FASTA sequences upon submission, but, if you prefer, you can also upload (optionally) the respective multi-GenBank file.

2. single FASTA files containing a particular complete or partial locus sequence (e.g., the traditionally used HA1 sequence of a virus representative of a particular clades/group). This can be used in "Extra Alignment/Phylogeny" projects.

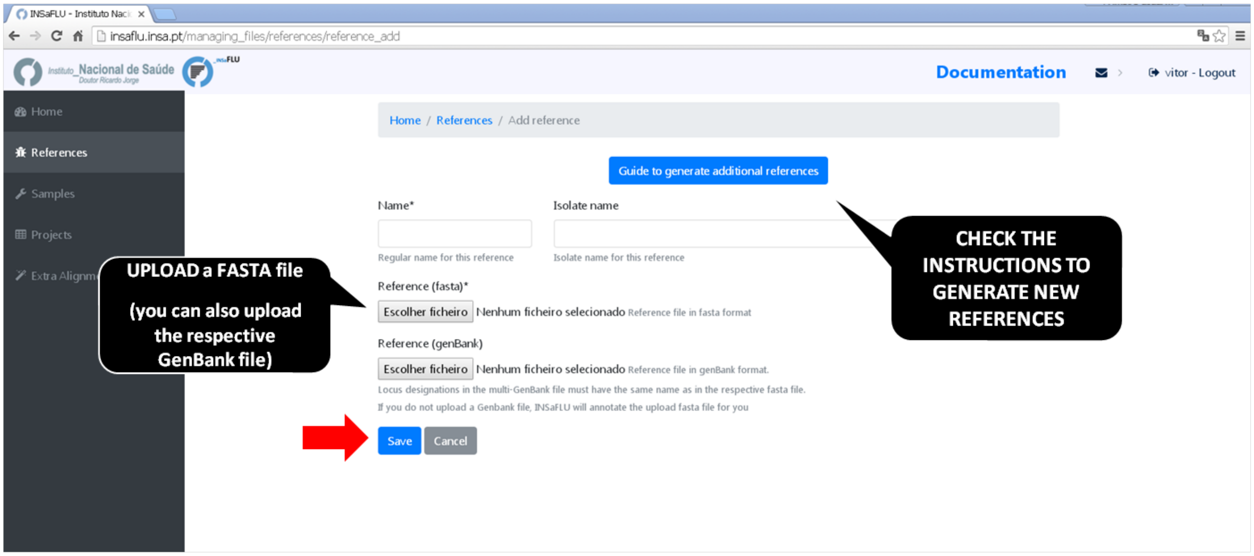

GUIDE TO GENERATE ADDITIONAL REFERENCE SEQUENCES

Please take this guide into account when generating additional reference sequences.

1. multi-FASTA files to be upload typically contain the set of reference sequences that constitute the influenza "whole-genome" sequence of a particular virus (e.g, the combination of the traditional 8 amplicons targeting the 8 eight influenza RNA segments). **Each individual sequence must have the precise size of each "intra-amplicon" target sequence that you capture by each one of the RT-PCR amplicons**. INSaFLU automatically annotates uploaded multi-FASTA sequences upon submission, but, if you prefer, you can also upload (optionally) the respective multi-GenBank file.

2. you may generate your multi-FASTA files in order to fit your amplicon schema by simply adjusting the whole-genome sequences available for download at INSaFLU or at influenza-specific sequence repositories, such as the Influenza Research Database (https://www.fludb.org), NCBI Influenza Virus Resource (https://www.ncbi.nlm.nih.gov/genomes/FLU/Database/nph-select.cgi?go=database) and EpiFLU/GISAID (https://www.gisaid.org/).

3. (multi) FASTA format is widely applied to save either nucleotide sequences or peptide sequences. An easy way to handle/generate multi-FASTA files is by opening a text file (e.g., NOTEPAD) and paste individual sequences after each header line. The FASTA IDs (after the '>' character) represent the individual sequence names. For the sake of simplicity, you may designate each sequence as 1, 2, 3, 4, 5, 6 , 7 and 8 (see example), following the traditional influenza segments order (keeping this numerical order is advisable). At the end, you just have to save the multi-FASTA file as ".fasta" (please avoid symbols or blank spaces in the file names).

   example: `A_H3N2_A_Perth_16_2009.fasta`

4. INSaFLU requires reference sequences exclusively composed by non-degenerate bases (i.e. A, T, C, or G). As such, please ensure that all degenerated bases (e.g., R, Y, M, K, S and W) are replaced by non-degenerate sequences before uploading. The choice of the base used in the replacement (e.g., "A" or "G" when replacing an "R") has no impact on the analysis. It simply means that mutations falling in the replaced nucleotide position will be reported taking into account the reference base selected.

3.2.3 Explore your Sample and Reference databases

Samples menu displays all information for all loaded samples (Samples' names in your account must be unique).

Upon submission, INSaFLU automatically updates samples' information with reads quality and typing data (automate bioinformatics pipeline modules "Read quality analysis and improvement" and Type and sub-type detection"; see Data

analysis in the Documentation).

Just explore the "More info" icon next to each sample.

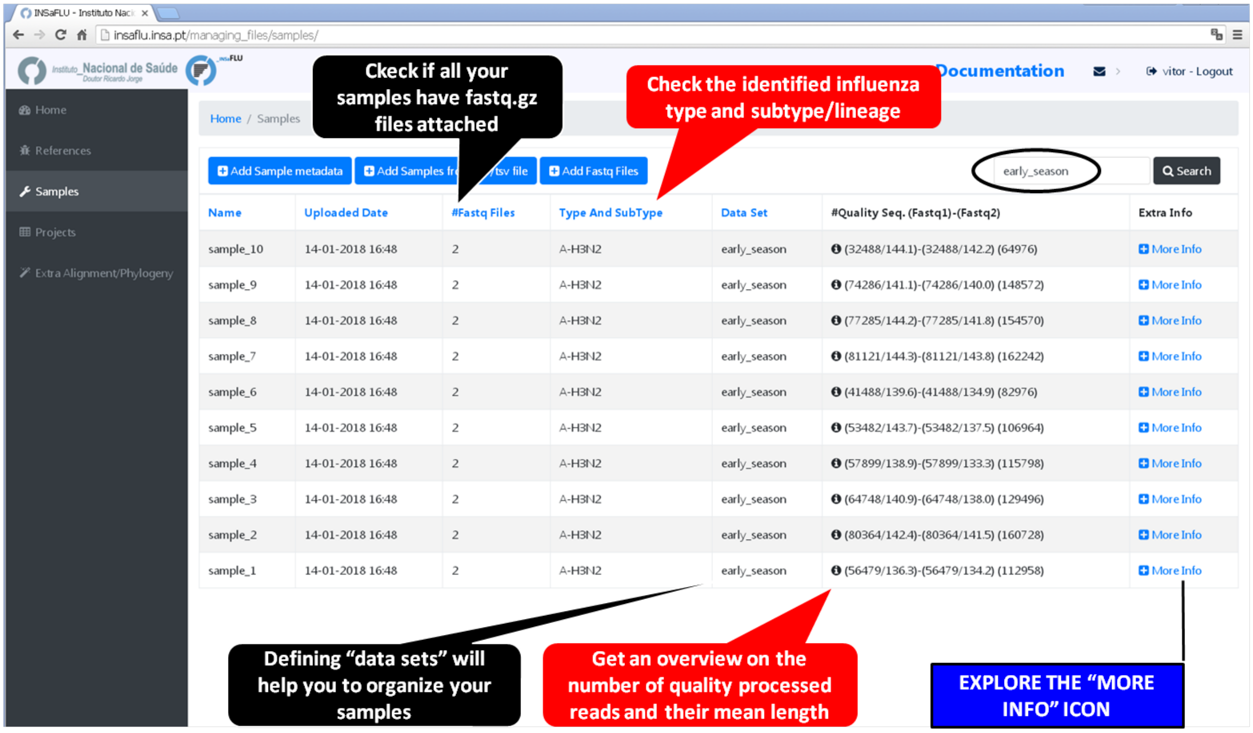

References menu displays all information for all reference sequences available at your confidential session.

Both FASTA and GenBank files can be downloaded by clicking on the displayed links.

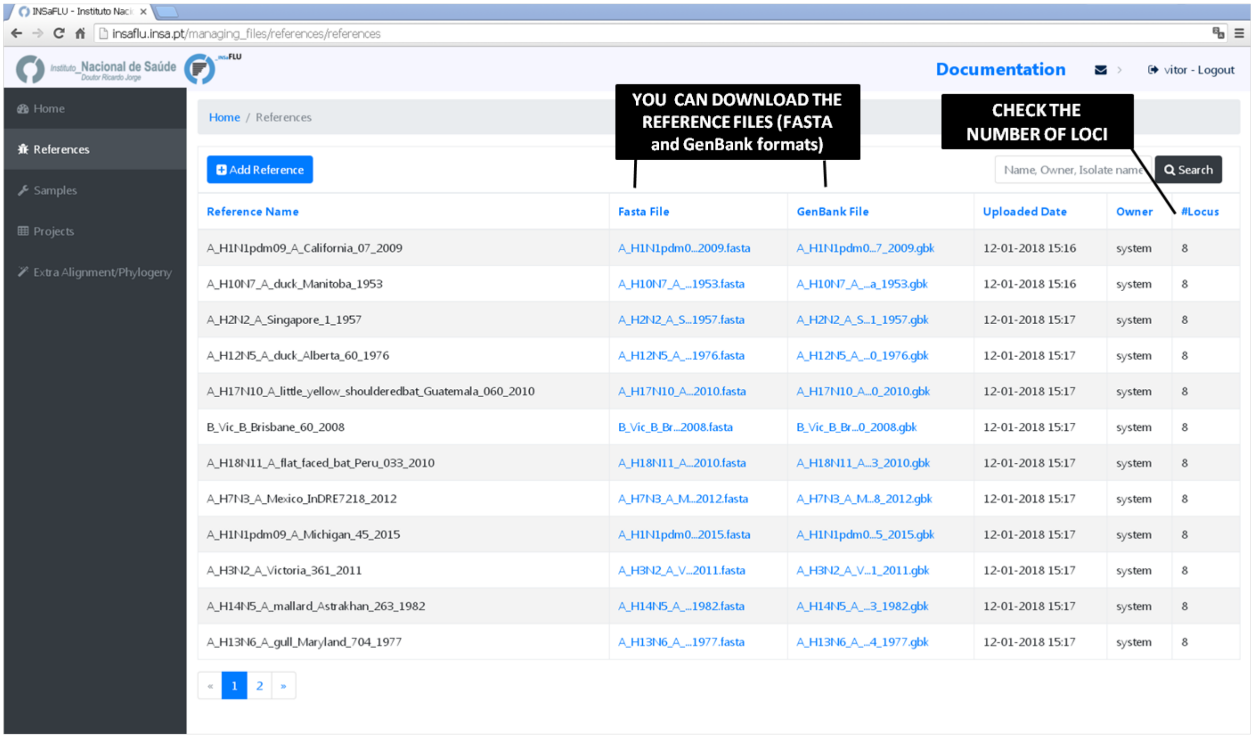

References:

- Zhou B, Donnelly ME, Scholes DT, St George K, Hatta M, Kawaoka Y, Wentworth DE. 2009. Single-reaction genomic amplification accelerates sequencing and vaccine production for classical and Swine origin human influenza a viruses. J Virol, 83:10309-13.
- Zhou B, Lin X, Wang W, Halpin RA, Bera J, Stockwell TB, Barr IG, Wentworth DE. 2014. Universal influenza B virus genomic amplification facilitates sequencing, diagnostics, and reverse genetics. J Clin Microbiol, 52:1330-1337.
- Zhou B, Wentworth DE. 2012. Influenza A virus molecular virology techniques. Methods Mol Biol, 865:175-92.

3.3 Project creation and scaling-up

One of the main goals of INSaFLU is to make data integration completely flexible and scalable in order to fulfill the analytical demands underlying laboratory surveillance throughout each flu epidemics. As such, INSaFLU allows users to create several projects (each one including multiple user-selected samples) and add more samples to each one as needed. In a dynamic manner, project outputs (e.g., gene- and genome-based alignments and phylogenetic trees) are automatically re-build and cumulatively updated as more samples are added to each project. The outputs are provided to be compatible with multiple downstream applications.

3.3.1 Creating and scaling-up a project

Within the *Projects* menu:

1. Go to *Projects* menu and choose *Create project*

You are encouraged to create "umbrella" projects, such as projects enrolling same sub-type viruses from the same season that will be compared with the vaccine reference virus for a given flu season.

You can designate the projects so that the name easily indicates the combination "virus sub-type/season/reference" (e.g. **A_H3N2_2017_18_vaccine_ref**)

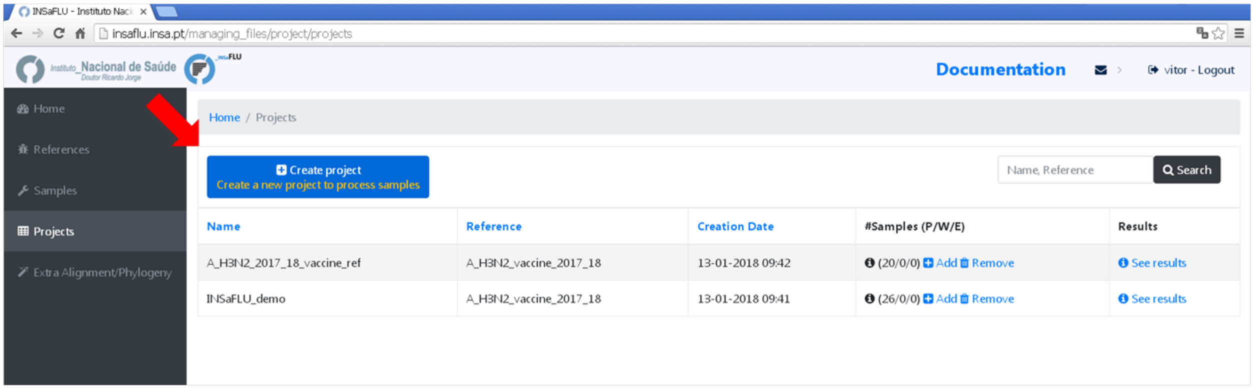

2. Choose a *Project Name*, select a *Reference sequence* and *Save*

Important: You should select a reference sequence (e.g., the vaccine strain from the current influenza season) that fits both your amplicon design (i.e., a multi-fasta file containing the set of reference sequences with the precise size of each "intra-amplicon" target sequence that you capture by each one of the RT-PCR amplicons) and the set of samples

that will be compared (e.g., same sub-type viruses from the same season to be compared with the vaccine reference virus).

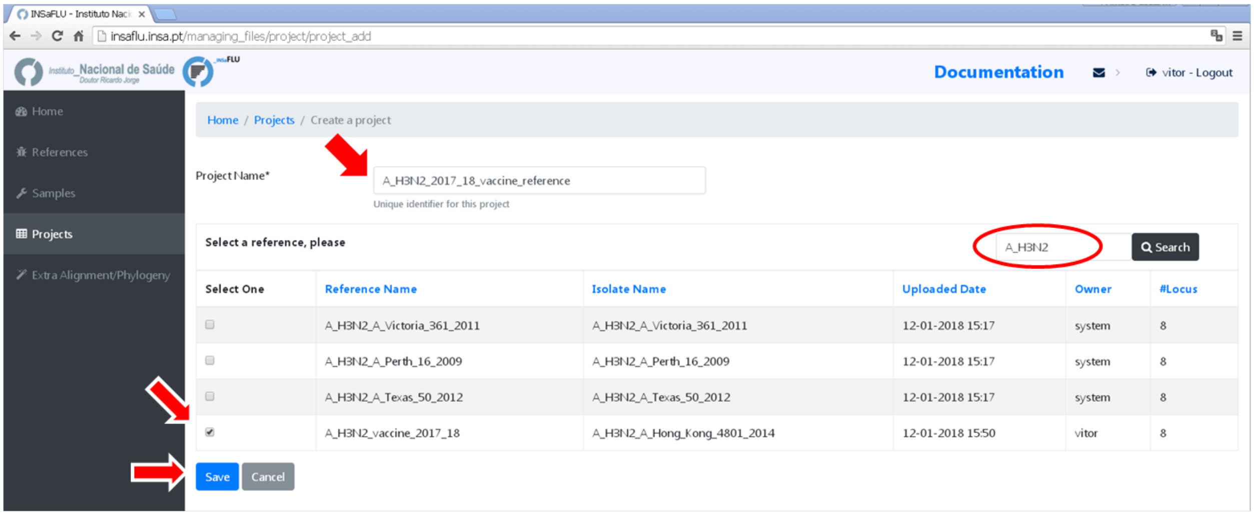

3. Add the samples to be included in the project

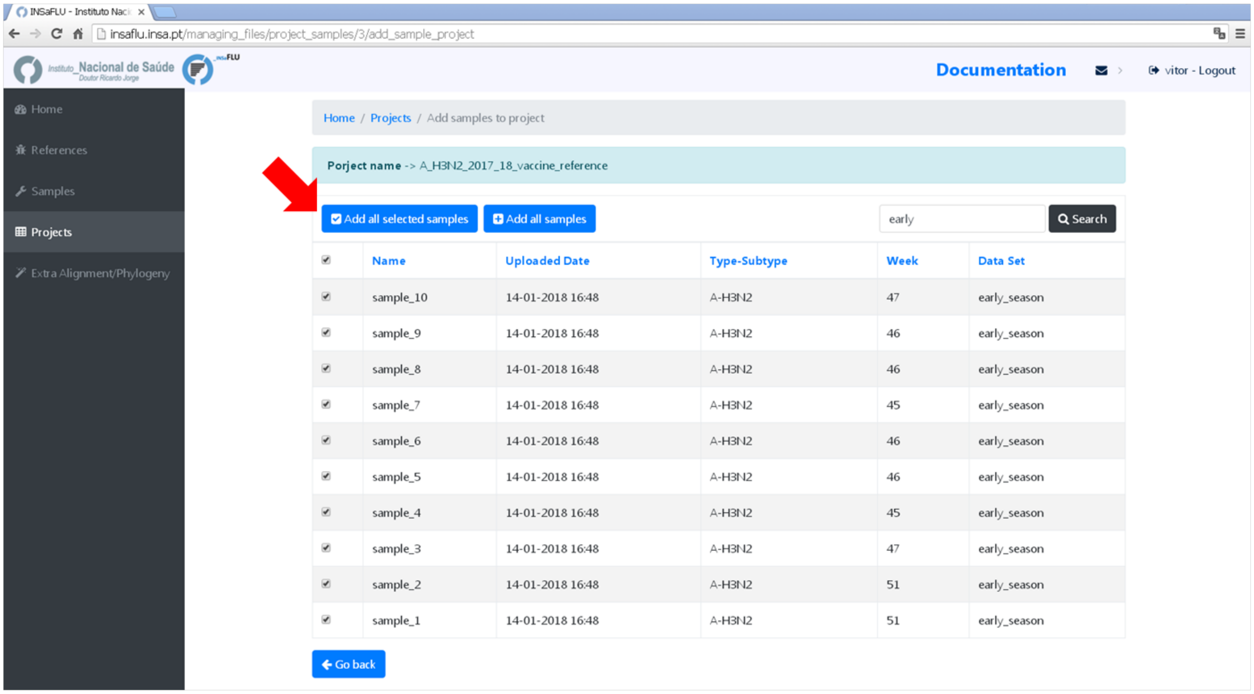

Samples are processed immediately upon selection, so, at this time, users may start monitoring the Project progress by checking the number of samples in the following status: Processed (P); Waiting (W) and Error (E).

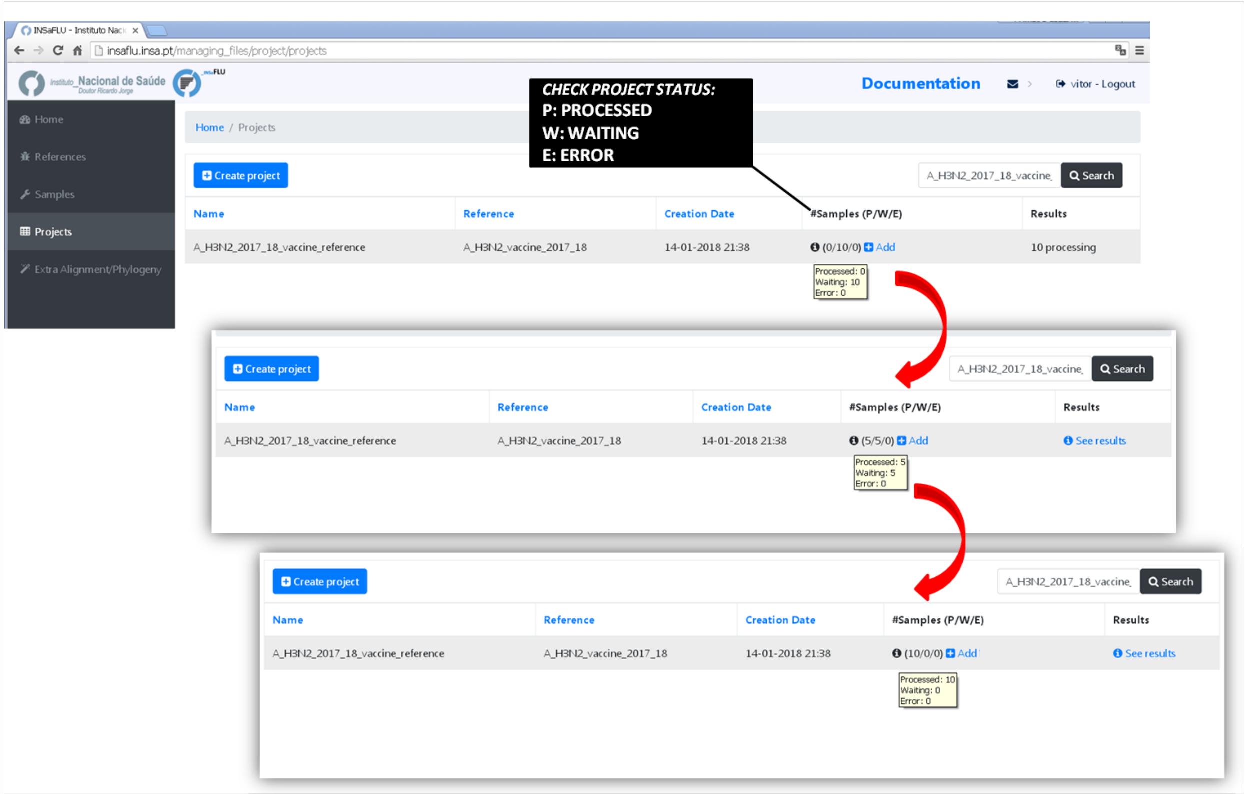

4. Scale-up your project.

You may add more samples to your **Project** project at any time.

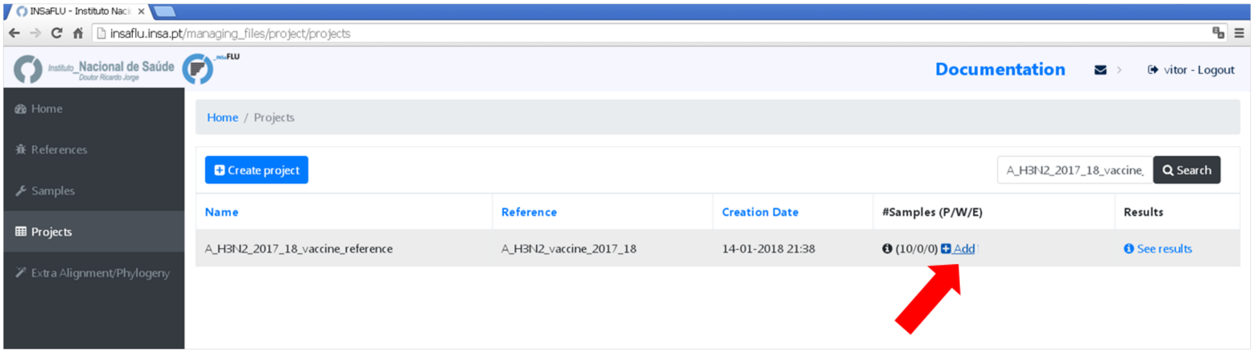

5. Remove samples from your project.

You may want to remove some samples from your project (e.g., for exclusively keeping samples with success for all 8 locus)

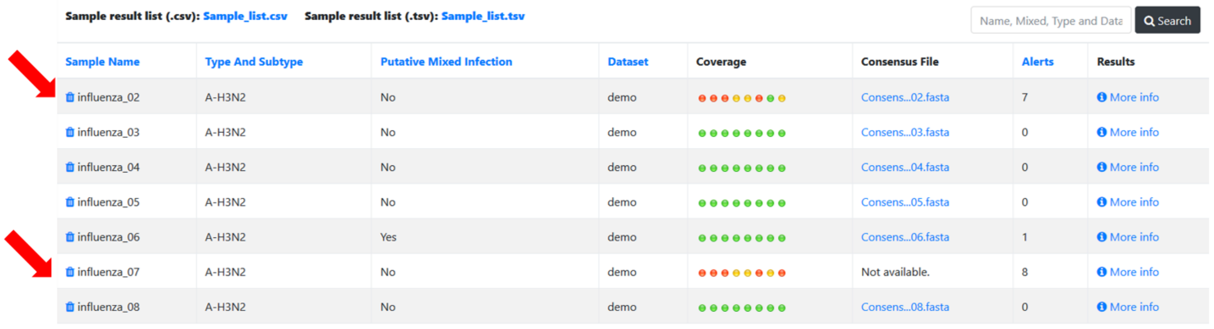

Sample result list (.csv): Sample_list.csv   Sample result list (.tsv): Sample_list.tsv

Sample Name	Type And Subtype	Putative Mixed Infection	Dataset	Coverage	Consensus File	Alerts	Results
influenza_02	A-H3N2	No	demo		Consens...02.fasta	7	More info
influenza_03	A-H3N2	No	demo		Consens...03.fasta	0	More info
influenza_04	A-H3N2	No	demo		Consens...04.fasta	0	More info
influenza_05	A-H3N2	No	demo		Consens...05.fasta	0	More info
influenza_06	A-H3N2	Yes	demo		Consens...06.fasta	1	More info
influenza_07	A-H3N2	No	demo		Not available.	8	More info
influenza_08	A-H3N2	No	demo		Consens...08.fasta	0	More info

3.3.2 Monitoring Projects' progress

INSaFLU projects are automatically run upon creation. So, at this time, users may start monitoring the Project progress by checking the number of samples in the following status: Processed (P); Waiting (W) and Error (E).

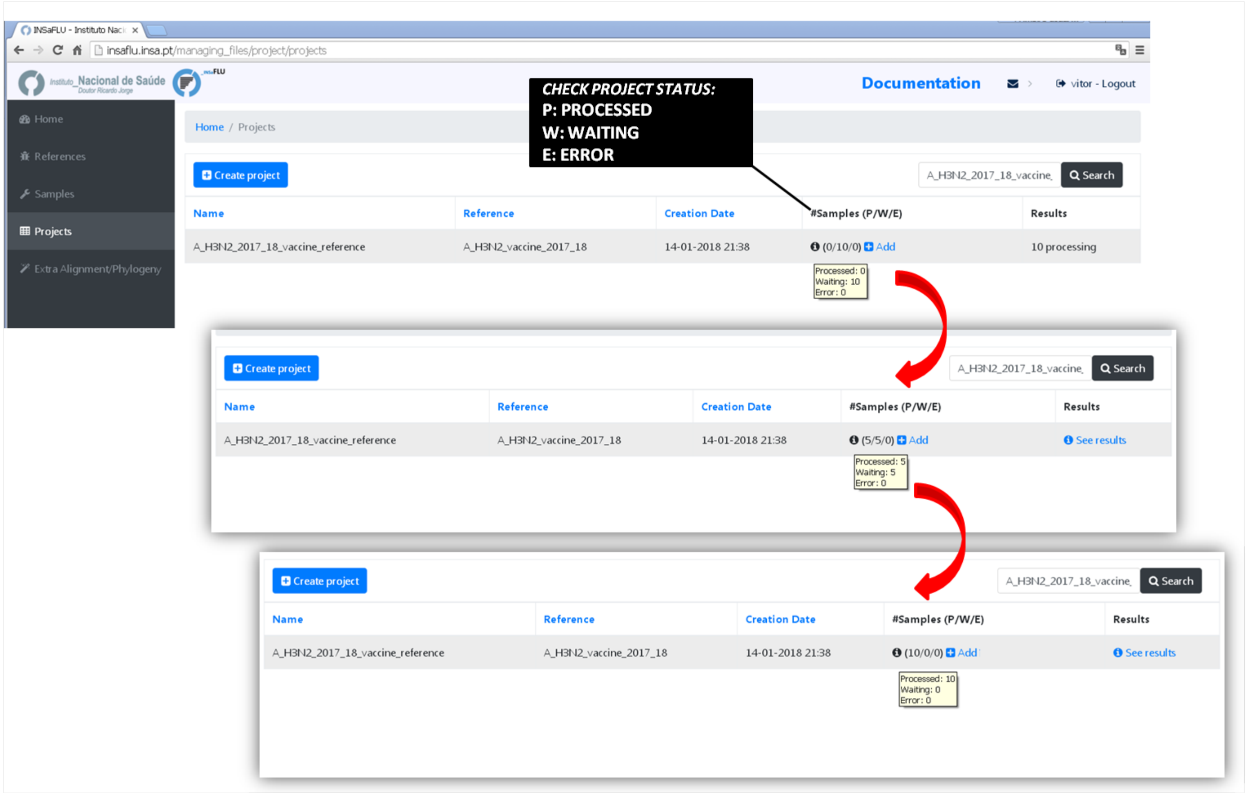

3.4 Data analysis

INSaFLU relies on a multi-software bioinformatics pipeline that will be under continuous development and improvement not only to enrich it with new features, but also to continuously shape the protocol to the best methodological advances in the field.

The current software settings, which were chosen upon intensive testing, are described below. For additional details

about the bioinformatics pipeline, please visit the INSaFLU github account: https://github.com/INSaFLU/INSaFLU (more information for each software can also be found in the official repositories; links are also provided below).

The bioinformatics pipeline developed and implemented in the INSaFLU web platform currently consists of 6 core steps (see WorkFlow) yielding multiple graphical, and sequence outputs (see *Output visualization and download* menu for details)

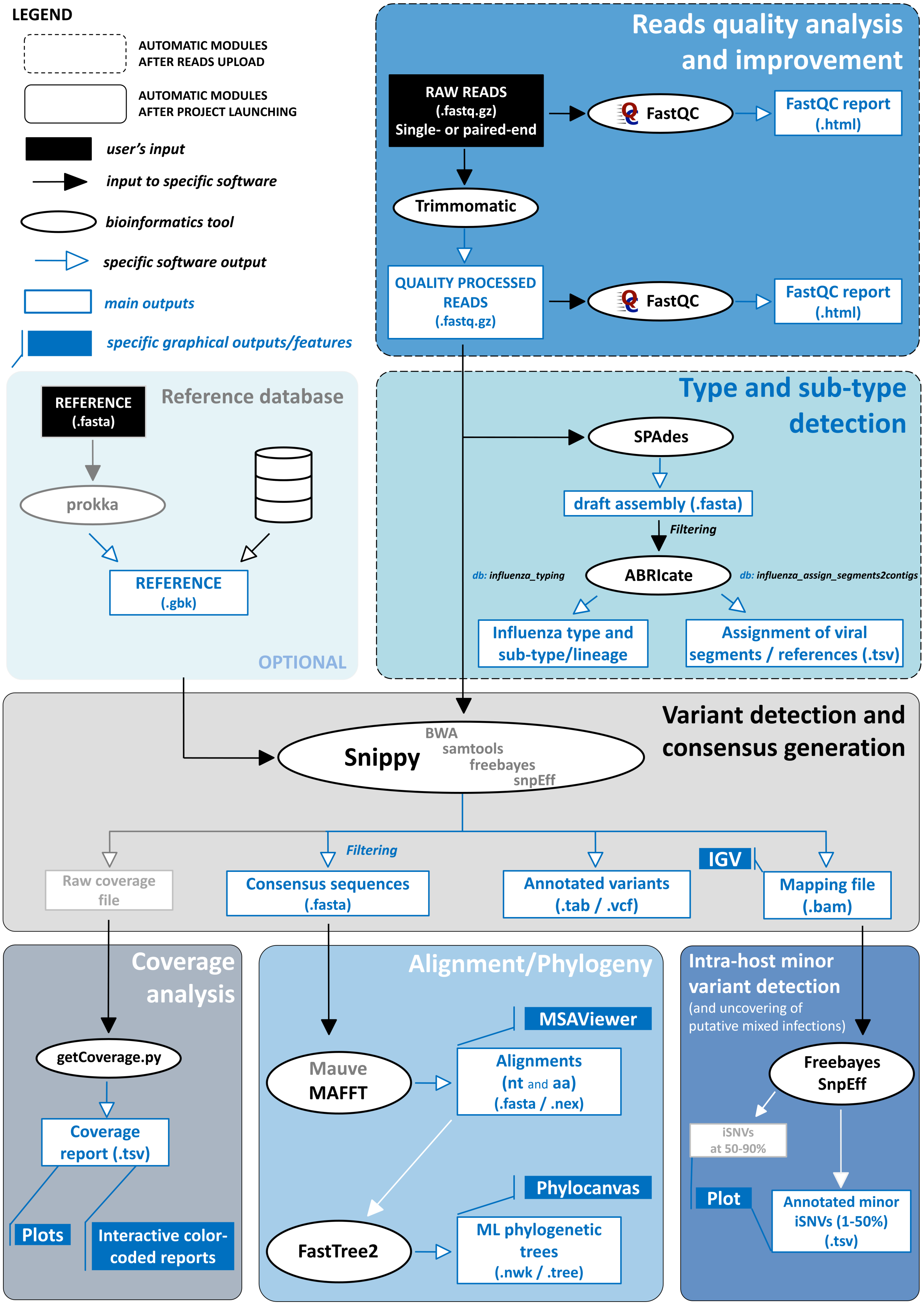

LEGEND
AUTOMATIC MODULES AFTER READS UPLOAD
AUTOMATIC MODULES AFTER PROJECT LAUNCHING
user's input
input to specific software
bioinformatics tool
specific software output
main outputs
specific graphical outputs/features
Reads quality analysis and improvement
RAW READS (.fastq.gz) Single- or paired-end
FastQC
FastQC report (.html)
Trimmomatic
QUALITY PROCESSED READS (.fastq.gz)
FastQC
FastQC report (.html)
Reference database
REFERENCE (.fasta)
prokka
REFERENCE (.gbk)
OPTIONAL
Type and sub-type detection
SPAdes
draft assembly (.fasta)
Filtering
db: influenza_typing
ABRIcate
db: influenza_assign_segments2contigs
Influenza type and sub-type/lineage
Assignment of viral segments / references (.tsv)
Variant detection and consensus generation
Snippy
BWA
samtools
freebayes
snpEff
Raw coverage file
Filtering
Consensus sequences (.fasta)
Annotated variants (.tab / .vcf)
IGV
Mapping file (.bam)
Coverage analysis
getCoverage.py
Coverage report (.tsv)
Plots
Interactive color-coded reports
Alignment/Phylogeny
Mauve
MAFFT
MSAViewer
Alignments (nt and aa) (.fasta / .nex)
FastTree2
Phylocanvas
ML phylogenetic trees (.nwk / .tree)
Intra-host minor variant detection
(and uncovering of putative mixed infections)
Freebayes
SnpEff
iSNVs at 50-90%
Plot
Annotated minor iSNVs (1-50%) (.tsv)

INSaFLU bioinformatics pipeline workflow

3.4.1 Bioinformatics pipeline

Modules (Description, Current software versions and settings)

Read quality analysis and improvement

Description

This step takes the input single- or paired-end reads (fastq.gz format) and produces Trimmomatic-derived quality processed reads, as well as FastQC quality control reports for each file, before and after quality improvement. This module is automatically run upon reads upload (i.e., no user intervention is needed).

Software version/settings

Note: **FastQC** (https://www.bioinformatics.babraham.ac.uk/projects/fastqc/) (version 0.11.5; date 15.01.2018)

input: single- or paired-end reads (fastq.gz format) (e.g., sample_L001_R1_001.fastq.gz and sample_L001_R2_001.fastq.gz for Illumina technology reads)

—nogroup option: all reports will show data for every base in the read.

Trimmomatic (http://www.usadellab.org/cms/index.php?page=trimmomatic) (version 0.27; date 15.01.2018)

input: single- or paired-end reads (fastq.gz format) (e.g., sample_L001_R1_001.fastq.gz and sample_L001_R2_001.fastq.gz for Illumina paired-end reads)

SLIDINGWINDOW: perform a sliding window trimming, cutting once the average quality within the window falls below a threshold (SLIDINGWINDOW:5:20, where 5 refers to window and 20 to the minimum average quality)

LEADING: cut bases off the start of a read, if below a threshold quality (LEADING:3). This will allow discarding bases with very quality or N bases (quality score of 2 or less).

TRAILING: cut bases off the end of a read, if below a threshold quality (TRAILING:3). This will allow discarding bases with very quality or N bases (quality score of 2 or less).

MINLEN: drop the read if it is below a specified length (MINLEN:35)

TOPHRED33: Convert quality scores to Phred-33

Files between 50 - 300 MB are downsized to ~50 MB before analysis by randomly sampling reads using fastq-sample from fastq-tools package https://github.com/dcjones/fastq-tools (developed by Daniel C. Jones dcjones@cs.washington.edu)

Type and sub-type identification

Description

This module uses quality processed reads obtained through Trimmomatic analysis and performs a draft de novo assembly using SPAdes. The assemblies are subsequently screened (using ABRIcate) against two INSaFLU in house sequence markers databases:

1. "influenza_typing", which drives the discrimination of the influenza types A and B, all currently defined influenza A subtypes (18 hemagglutinin subtypes and 11 neuraminidase sub-types) and the two influenza B lineages (Yamagata and Victoria).

2. "influenza_assign_segments2contigs", which allows the automatic assignment of the assembled contigs to both the corresponding viral segments and to a related reference influenza virus.

The generated outputs (i.e., draft assemblies, the identified type and subtype/lineage and a table linking contigs to segments/references) are automatically provided upon reads upload (i.e., no user intervention is needed). INSaFLU flags samples as "putative mixed infections" if more than one type, HA or NA subtype or lineage is detected. In addition, specific alerts are generated if an incomplete type/subtype is assigned.

Software version/settings

Note: **SPAdes** (http://cab.spbu.ru/software/spades/) (version 3.11.1; date 15.01.2018)

–pe1-1 and –pe1.2 (for paired-end) or -s (for single-end data): define the input files, i.e, quality processed reads (e.g., sample_1P.fastq.gz and sample_2P.fastq.gz)

—only-assembler: runs assembly module only and does not perform reads correction

(contigs with k-mer coverage below '3' are discarded for subsequent ABRIcate analyses to avoid the classification of vestigial sequencer-derived contaminating sequences)

ABRIcate (https://github.com/tseemann/abricate) (version 0.8-dev; date 15.01.2018)

For type and subtype/lineage identification:

—db influeza_typing: the INSaFLU "influenza_tying" database includes a set of type- and sub-type/lineage-specific gene markers that ensure the discrimination of the influenza types A and B, all currently defined influenza A subtypes (18 hemagglutinin subtypes and 11 neuraminidase sub-types) and the two influenza B lineages (Yamagata and Victoria).

–minid: minimum DNA %identity (–minid 70)

—mincov: minimum DNA % coverage (–mincov 60)

For segments/references assignment:

—db influeza_assign_segments2contigs: this database includes segment sequence markers of several seasonal human influenza [including: i) post-pandemic (2009) vaccine/reference influenza A(H1N1)pdm2009, A(H3N2) and B viruses; ii) representative viruses of specific genetic groups/lineages/clades, as defined by International Health Authorities for each season)], as well as of avian influenza from several HA/NA subtypes (i.e., H1N1, H2N2, H5N1, H7N9, etc)

–minid: minimum DNA %identity (–minid 70)

—mincov: minimum DNA % coverage (–mincov 30)

Important note: Since the "influeza_assign_segments2contigs" database is naturally not as exhaustive as other databases (such as, NCBI, Fludb or EpiFLU/GISAID), users may need to run the draft assemblies in these databases (or associated tools, such as BLAST) for some purposes (e.g., to detect/confirm reassortments or to infer the closest reference sequence of each segment).

Latest lists of genetic markers can be downloaded here: `INSaFLU_current_genetic_markers_05_06_2018.xlsx`

Variant detection and consensus generation

Description

This key module takes advantage of the multisoftware tool Snippy (please visit the official repository to get details about each component; https://github.com/tseemann/snippy) to perform reference-based mapping, followed by

SNP/indel calling and annotation and generation of consensus sequences (quality processed reads obtained through Trimmomatic analysis are used as input). A reference sequence is selected for each project after uploading it or from the INSaFLU default reference database. Uploaded ".fasta" files are annotated using Prokka upon submission and automatically become available at the user-restricted reference database. Each project should ideally include viruses from the same type and sub-type/lineage (this typing data is automatically determined upon reads submission to INSaFLU).

Software version/settings

Note: **Prokka** (https://github.com/tseemann/prokka) (version 1.2; date 15.01.2018)

–kingdom: defines the Annotation mode (Viruses)

Snippy (https://github.com/tseemann/snippy) (version 3.2-dev - sligthly modified (details in https://github.com/INSaFLU/INSaFLU); date 15.01.2018)

–R1 (and –R2): define the reads files used as input, i.e, quality processed reads (e.g., sample_1P.fastq.gz and sample_2P.fastq.gz) obtained after Trimmomatic analysis

—ref: define the reference sequence selected by the users (.fasta or gbk format)

–mapqual: minimum mapping quality to allow (–mapqual 20)

—mincov: minimum coverage of variant site (–mincov 10)

–minfrac: minumum proportion for variant evidence (–minfrac 0.51)

Integrative Genomics Viewer (http://software.broadinstitute.org/software/igv/) (version 2.3.98; date 15.01.2018)

inputs: reference file (.fasta); mapping file (.bam; .bai)

Coverage analysis

Description

This module yields a deep analysis of the coverage for each per sample by providing the following data: mean depth of coverage per locus, % of locus size covered by at least 1-fold and % of locus size covered by at least 10-fold. The latter fits the minimum depth of coverage for variant calling applied by INSaFLU pipeline and constitutes the guide for consensus generation, i.e., consensus sequences (see Module "Variant detection and consensus generation") are exclusively provided for locus fulfilling the criteria of having 100% of their size covered by at least 10-fold. Depth of coverage plots are additionally generated and can be interactively viewed at INSaFLU.

Software version/settings

Note: **getCoverage.py** (https://github.com/monsanto-pinheiro/getCoverage) (version v1.1; date 15.01.2018)

-i: define the input files, i.e, the coverage files (.depth.gz) generated through Snippy

-r: define the reference sequence selected by the users (.fasta format)

-o: defines the output file name (tab-separated value)

Alignment/Phylogeny

Description

This module uses filtered nucleotide consensus sequences and performs refined nucleotide/protein sequence alignments and phylogenetic inferences. These outputs are automatically re-build and updated as more samples are added

to user-restricted INSaFLU projects, making continuous data integration completely flexible and scalable. This module can also be run independently over a set of user-selected sequences (e.g., circulating virus plus sequences of representative virus of specific genetic groups/clades/lineages), so that phylogenetic diversity of circulating viruses can be better evaluated and integrated in the frame of guidelines defined by supranational health authorities.

Software version/settings

Note: **MAUVE** (http://darlinglab.org/mauve/mauve.html) (version 2.4.0; date 15.01.2018)

progressiveMAUVE module (default settings): this algorithm is applied to perform primary draft alignments, and has the particular advantage of automatically concatenating multi-fasta input sequences during whole-genome alignments construction.

input file: filtered nucleotide consensus sequences for each sample, one per each amplicon target (which are , in general, influenza CDSs) and another for the whole-genome sequence (i.e., the set of sequence targeted by the amplicon-based NGS shema, which, in general, is the pool of main 8 influenza CDSs). xmfa to fasta conversion is carried out using "convertAlignment.pl" (https://github.com/lskatz/lyve-SET/blob/master/scripts/convertAlignment.pl

(default settings)

MAFFT (https://mafft.cbrc.jp/alignment/software/) (version 7.313; date 15.01.2018)

For nucleotide alignments:

input file: progressiveMAUVE-derived draft alignments (multifasta format), one per each locus and another for the whole-genome sequence

(default settings)

For amino acid alignments:

–amino: assume the sequences are in amino acid.

FastTree (http://www.microbesonline.org/fasttree/) (version 2.1.10 Double precision; date 15.01.2018)

Double-precision mode: suitable for resolving very-short branch lengths accurately (FastTreeDbl executable)

-nt: defines the input nucleotide alignment, which is a MAFFT-derived refined alignments (multifasta format). Alignments to be run include one per each locus and another for the whole-genome sequence.

—gtr: defines the Generalized time-reversible (GTR) model of nucleotide evolution (CAT approximation with 20 rate categories)

-boot: defines the number resample (-boot 1000)

Seqret EMBOSS tool (http://emboss.sourceforge.net/apps/release/6.6/emboss/apps/) (version 6.6.0.0; date 15.01.2018)

input file: nucleotide alignments in FASTA (.fasta) to be converted in NEXUS (.nex) format

MSAViewer (http://msa.biojs.net/) (latest; date 15.01.2018)

input files: consensus nucleotide alignments for each locus and for the consensus 'whole-genome' sequence (upon concatenation of all individual locus); and amino acid alignments for the encoded proteins

Phylocanvas (http://phylocanvas.org/) (version 2.8.1; date 15.01.2018)

input files: phylogenetic tree for each locus-specific nucleotide alignment and for the alignments of the 'whole-genome' sequences (upon concatenation of all individual locus)

Intra-host minor variant detection (and uncovering of putative mixed infections)

Description

This module uses mapping data for the set of samples from each user-restricted INSaFLU project and provides a list of minor intra-host single nucleotide variants (iSNVs), i.e., SNV displaying intra-sample frequency between 1- 50%. This output is automatically re-build and cumulatively updated as more samples are added to each INSaFLU project, making continuous data integration completely flexible and scalable. Plots of the proportion of iSNV at frequency at 1-50% (minor iSNVs) and at frequency 50-90% detected for each sample are also provided as mean to a guide the uncovering of putative mixed infections (exemplified in the Figure). INSaFLU flags samples as "putative mixed infections" based on intra-host SNVs if the following cumulative criteria are fulfilled: the ratio of the number of iSNVs at frequency at 1-50% (minor iSNVs) and 50-90% falls within the range 0,5-2,0 and the sum of the number of these two categories of iSNVs exceeds 20. Alternatively, to account for mixed infections involving extremely different viruses (e.g., A/H3N2 and A/H1N1), the flag is also displayed when the the sum of the two categories of iSNVs exceeds 100, regardless of the first criterion.

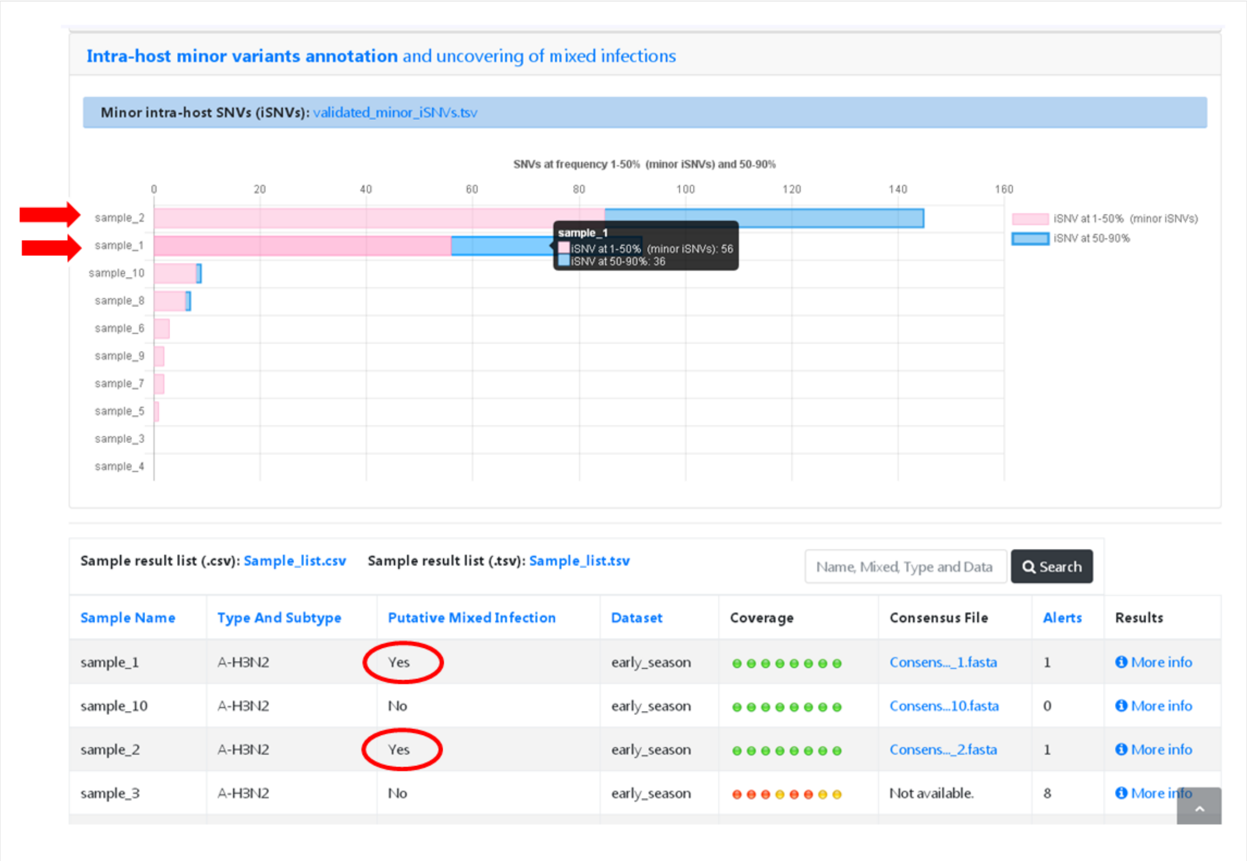

Software version/settings

Note: Freebayes (https://github.com/ekg/freebayes) (version v1.1.0-54-g49413aa; date 15.01.2018)

–min-mapping-quality: excludes read alignments from analysis if they have a mapping quality less than Q (–min-mapping-quality 20)

—min-base-quality: excludes alleles from iSNV analysis if their supporting base quality is less than Q (–min-base-quality 20)

–min-coverage: requires at least 100-fold of coverage to process a site (–min-coverage 100)

—min-alternate-count: require at least 10 reads supporting an alternate allele within a single individual in order to evaluate the position (–min-alternate-count 10)

–min-alternate-fraction: defines the minimum intra-host frequency of the alternate allele to be assumed (–min-alternate-fraction 0.01). This frequency is contingent on the depth of coverage of each processed site since min-alternate-count is set to 10, i.e., the identification of iSNV sites at frequencies of 10%, 2% and 1% is only allowed for sites with depth of coverage of at least 100-fold, 500-fold and 1000-fold, respectively.

3.5 Output Visualization and Download

Upon project's launching, you can start exploring the diverse INSaFLU outputs, which include:

- **sample-specific outputs** (such as, mapping files, variants annotation and consensus sequences)
- **project outputs** (such as, nucleotide/amino acid alignments and phylogenetic trees).

Outputs are organized by the dynamic "expand-and-collapse" panels that allow you a user-friendly visualization/download of all graphical, text and sequence output data. The following table provides an overview on all INSaFLU outputs organized by bioinformatics module:

`INSaFLU_current_outputs_14_05_2018.xlsx`

While navigating through INSaFLU menus, you will find which main software (including versions and settings) were used to generate outputs.

3.5.1 Navigate through sample-specific outputs

Explore the *Samples* menu

This tab displays all information for all loaded samples.

A. Go to *Samples* menu and check the *reads' quality reports and typing data*

Just after samples' metadata and NGS data submission, INSaFLU automatically updates samples' information with reads quality and typing data .

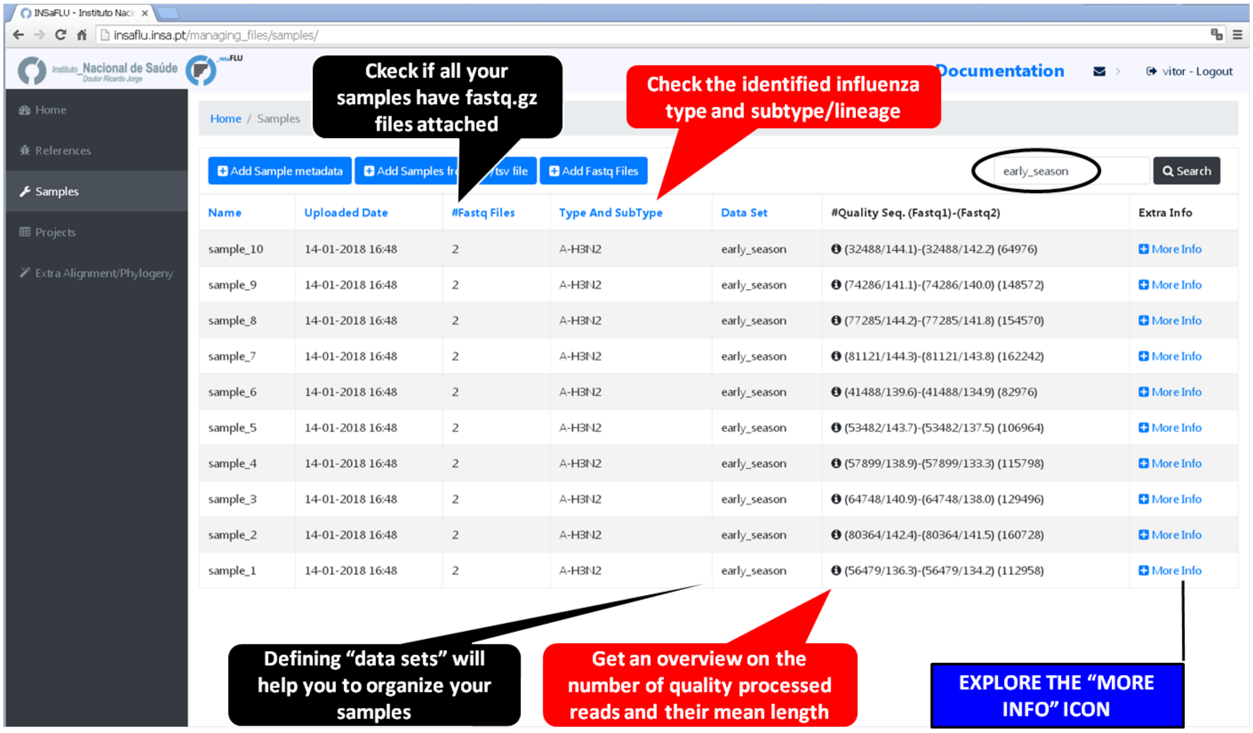

B. Go to *Samples* menu and explore the *'More info' icon next to each sample*.

By clicking on the 'More info' icon next to each sample, you can get an overview on the specific sample metadata and explore:

- **FastQC graphical quality reports for raw read files**

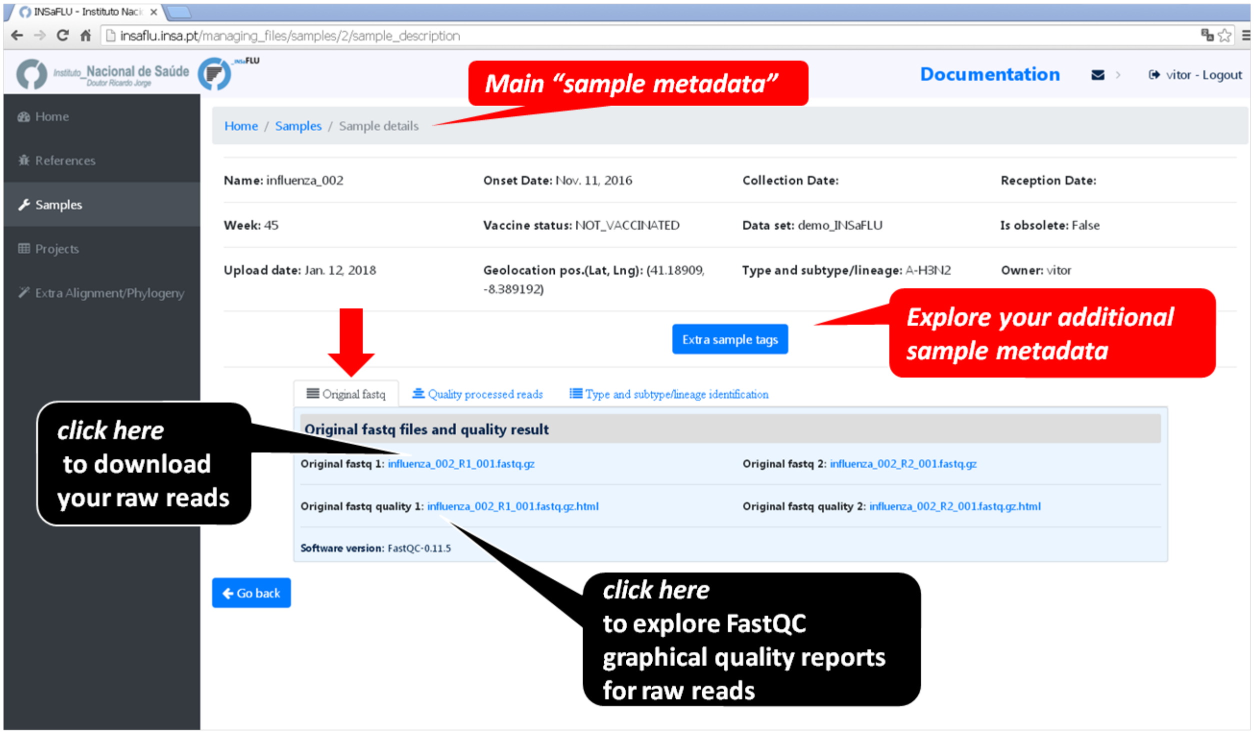

Click on ".html" files and explore each one of the FastQC "Analysis modules" - please consult https://www.bioinformatics.babraham.ac.uk/projects/fastqc/Help/3%20Analysis%20Modules/ for details]

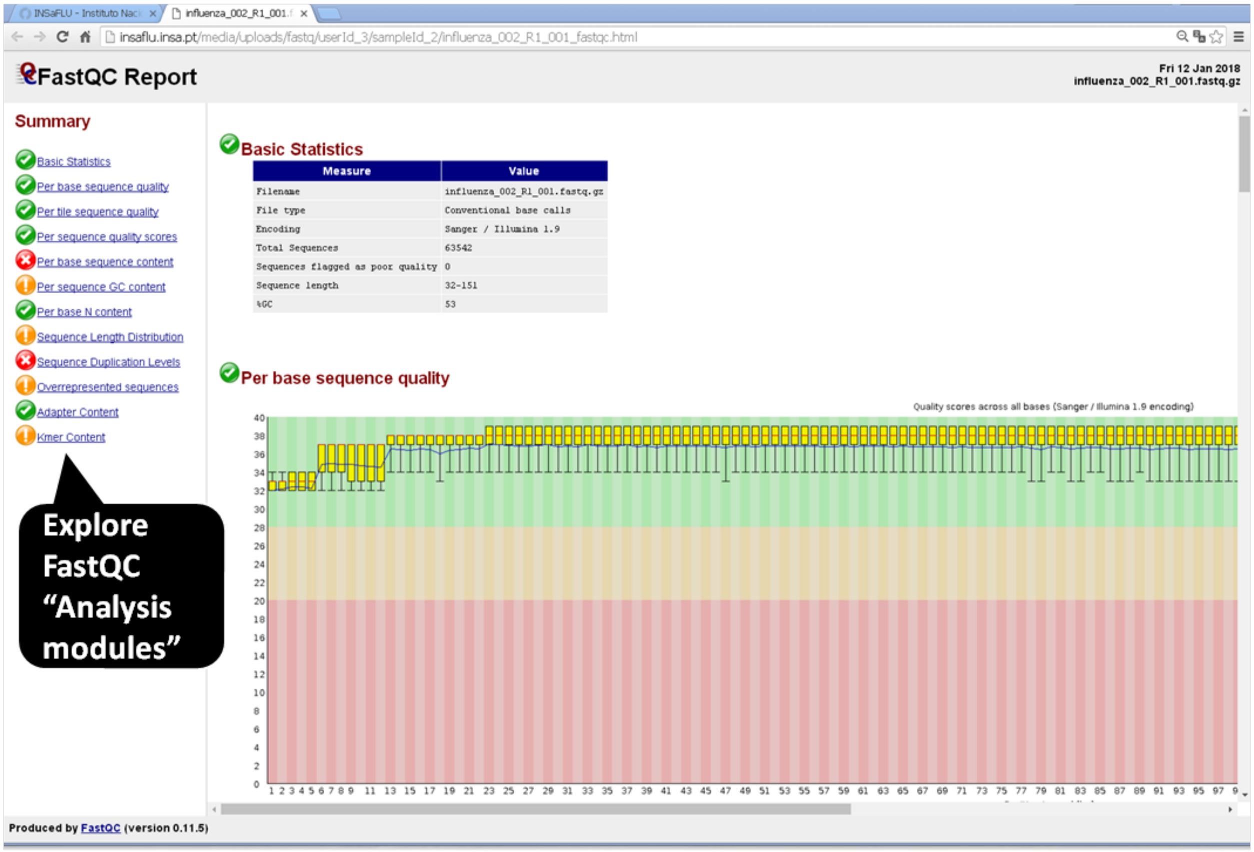

- **FastQC graphical quality reports for quality processed read files**

Click on ".html" files and explore each one of the FastQC "Analysis modules" - please consult https://www.bioinformatics.babraham.ac.uk/projects/fastqc/Help/3%20Analysis%20Modules/ for details]

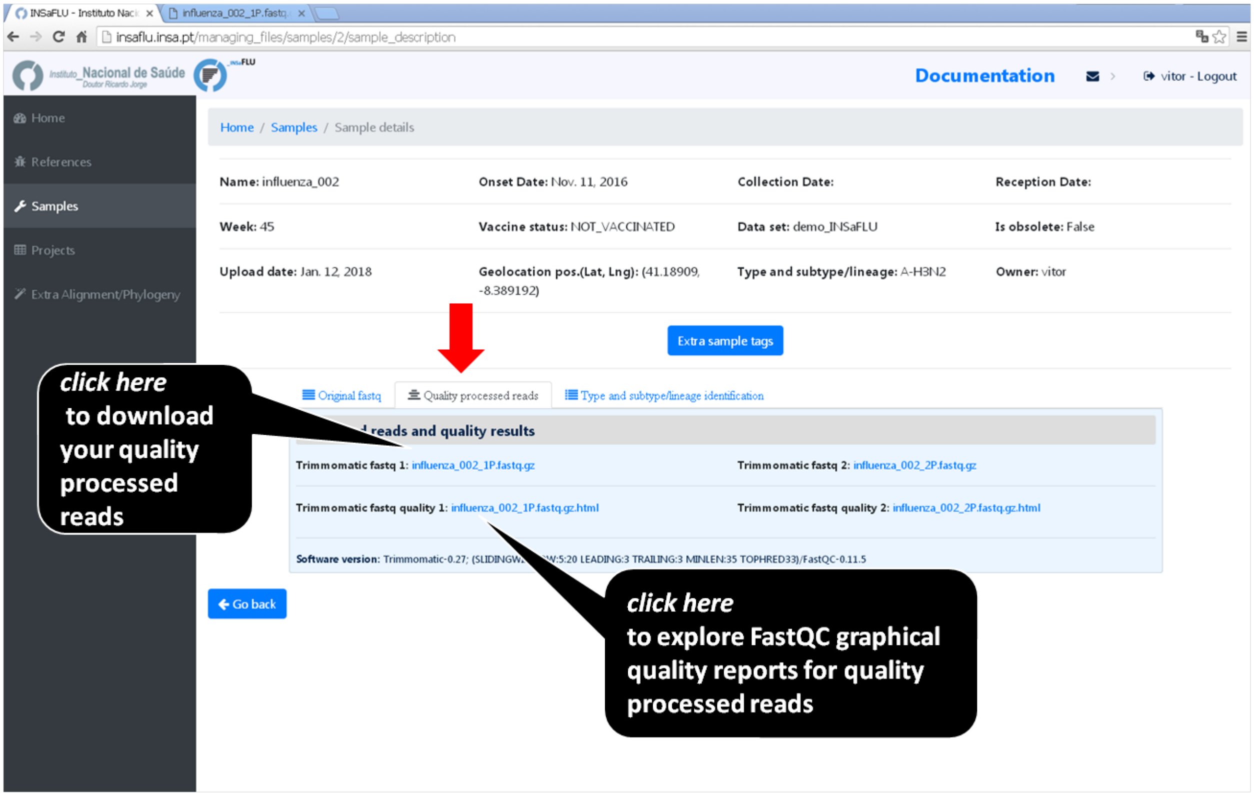

- **Typing and subtyping data**

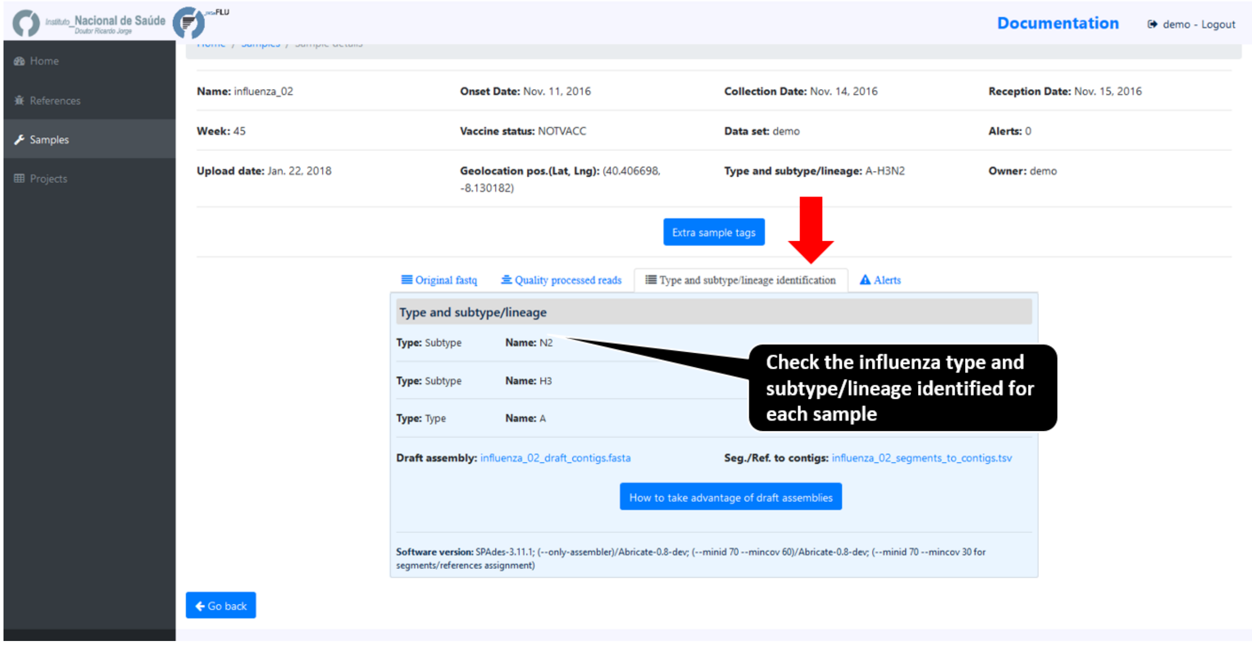

Note:

- INSaFLU allows the discrimination of the influenza types A and B, all currently defined influenza A subtypes (18 hemagglutinin subtypes and 11 neuraminidase sub-types) and the two influenza B lineages (Yamagata and Victoria).

- INSaFLU flags samples as "putative mixed infections" if more than one type, HA or NA subtype or lineage is detected. In addition, specific alerts are generated if an incomplete type/subtype is assigned.

- Assignment of viral segments/references to draft contigs

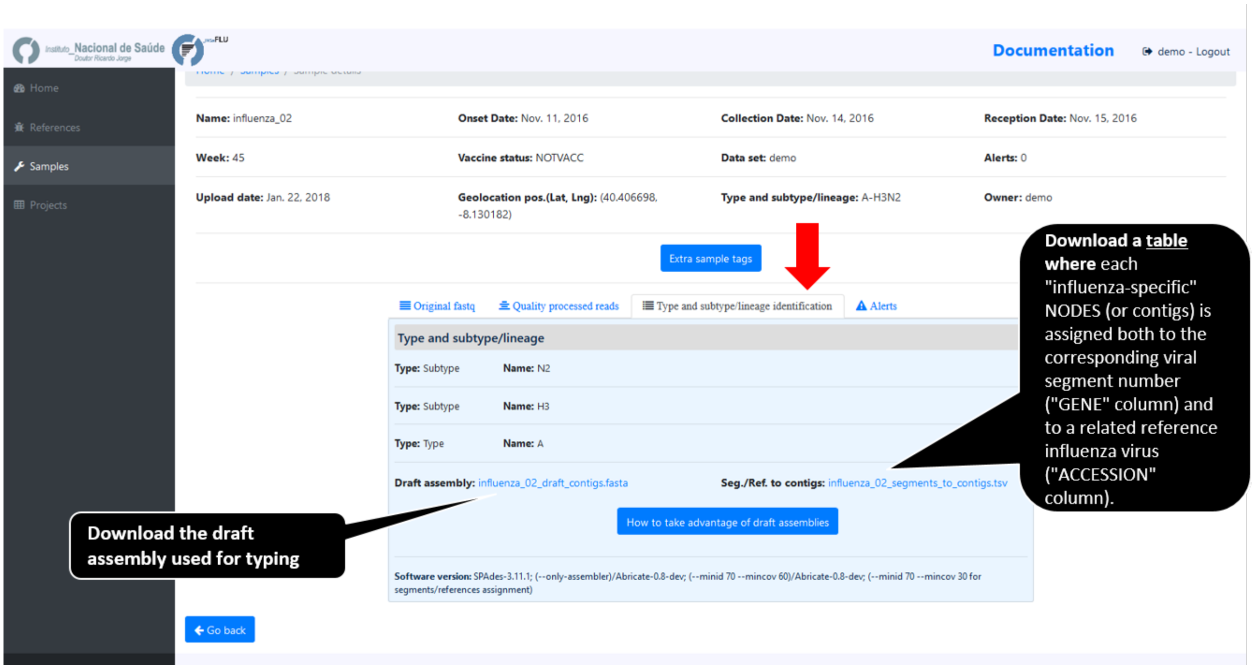

Note:

- INSaFLU performs automatic assignment of each assembled influenza-specific NODE (or contig) to both the corresponding viral segments and to a related reference influenza virus.
  - Outputs of this module (i.e., draft assemblies, the identified type and subtype/lineage and a table linking contigs to segments/references) can be visualize or downloaded here.

Explore the *Projects* menu

This tab lists all your projects.

Multiple sample-specific outputs (see below) are generated when a given sample is processed within an specific Project.

A. Go to *Projects* menu, and click on "See results" to explore outputs of a given project

Below the dynamic 'expand-and-collapse' panels, you can explore a table that contains multiple sample-specific outputs generated for each sample in a given project, including:

- **Type and subtype/lineage**
- **Putative mixed infection**
- **Coverage report per locus** (interactive color-coded statistics and plots of the depth of coverage throughout each locus sequence)

- **Consensus sequence for the pool of loci**

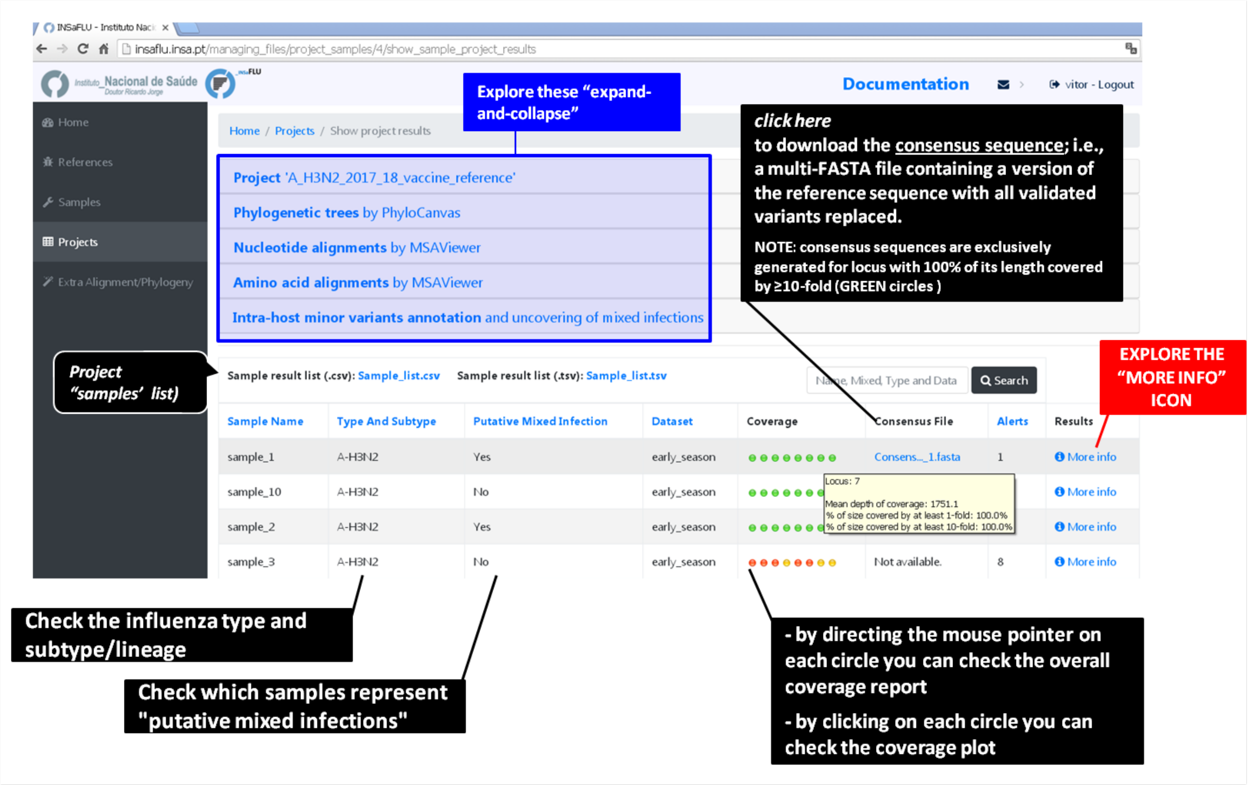

Important: COVERAGE COLOR CODE:

GREEN: % of locus size covered by at least 1-fold = 100 % and % of locus size covered by at least 10-fold = 100%

YELLOW: % of locus size covered by at least 1-fold = 100 % and % of locus size covered by at least 10-fold < 100%

RED: % of locus size covered by at least 1-fold < 100 % and % of locus size covered by at least 10-fold < 100%

By clicking on each one of the color-coded circles, you can explore locus-specific plots of the depth of coverage

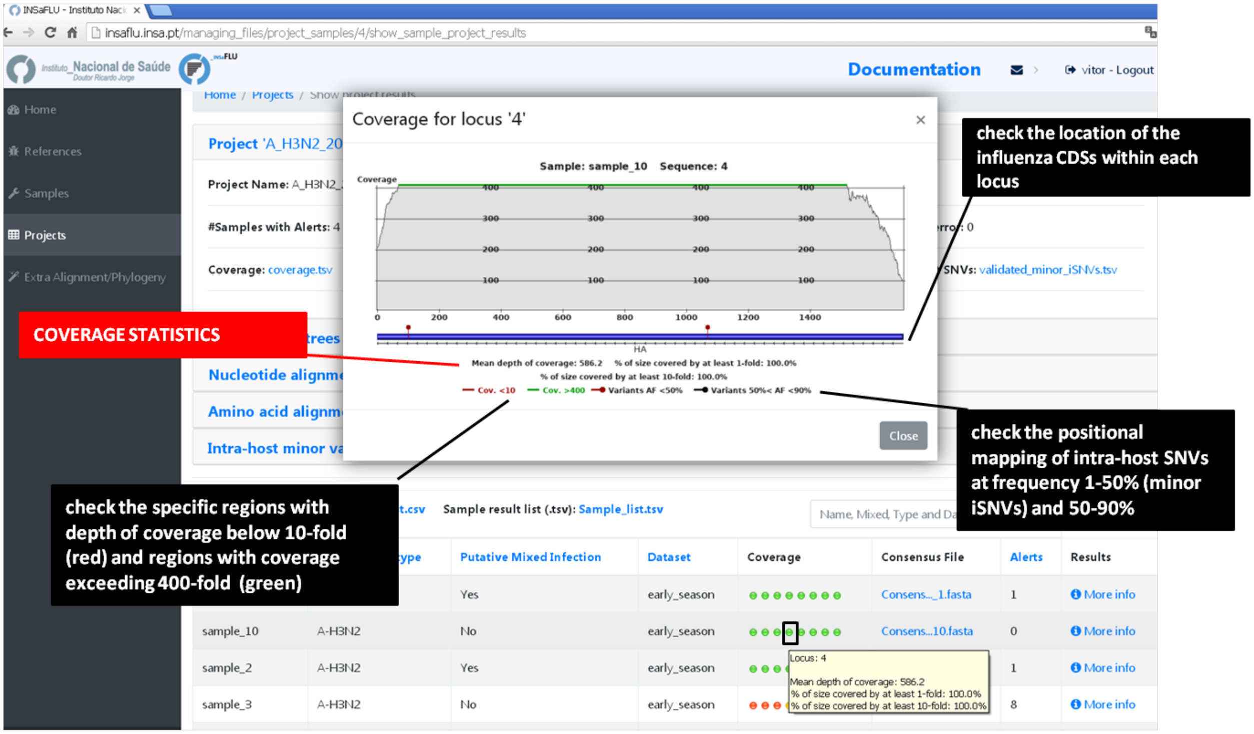

B. Go to *Projects* menu, click on "See results" and explore the "More info" icon next to each sample

By clicking on the 'More info' icon next to each sample, you can get an overview on the specific sample metadata and additionally download/explore:

- **Type and subtype/lineage**
- **Mapping file**
- **Consensus sequence for the pool of loci**
- **Annotated variants (SNPs and indels)**

Warning:

- Validated variants falling within loci not fully covered with 10-fold (color-coded as yellow or red) are still included in the "validated_variants" list (these cases are labeled in the table column "VARIANTS in INCOMPLETE LOCUS" as YES), so that users can still retrieve valuable and reliable data (e.g., specific epitope and antiviral drug resistance mutations) from samples with borderline coverage.
- Consensus sequences are exclusively generated for individual locus with 100% of its length covered by at least 10-fold (GREEN code in the grapgical coverage report)

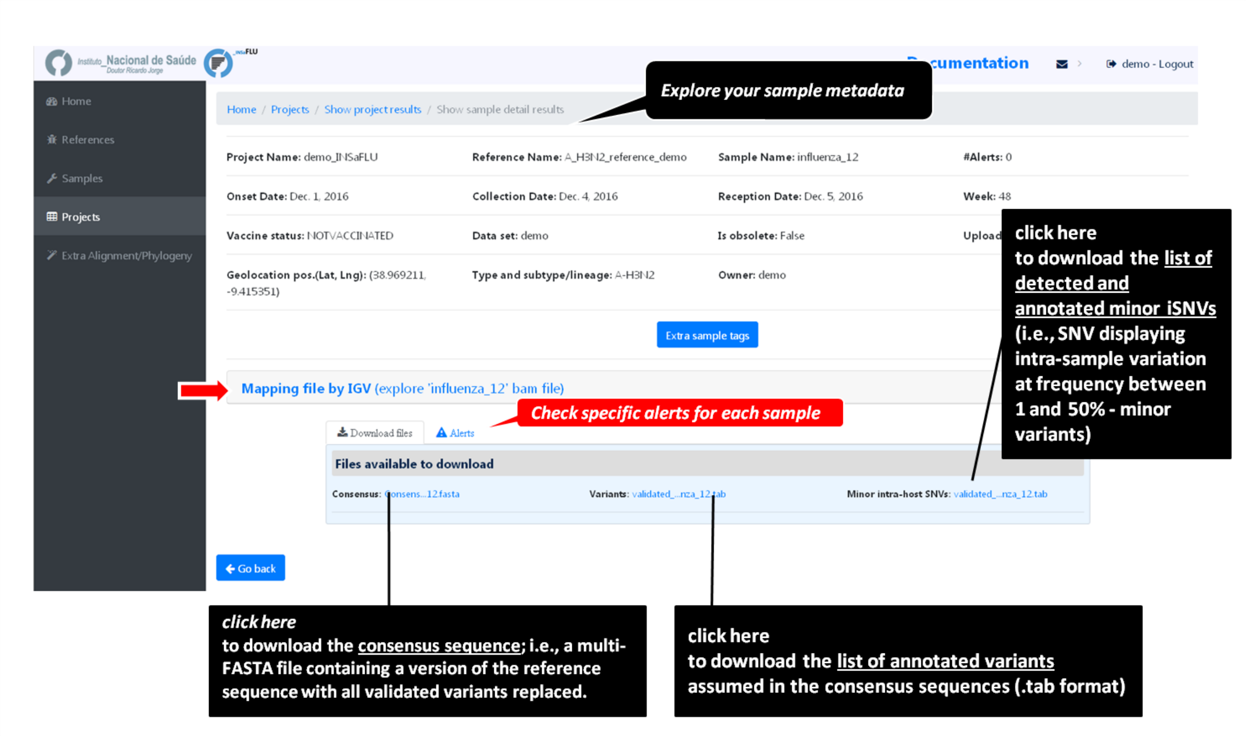

By clicking on "Mapping file by IGV (Explore 'sample.bam' file), you can finely inspect the mapped reads (and variants) using the Integrative Genomics Viewer (IGV)

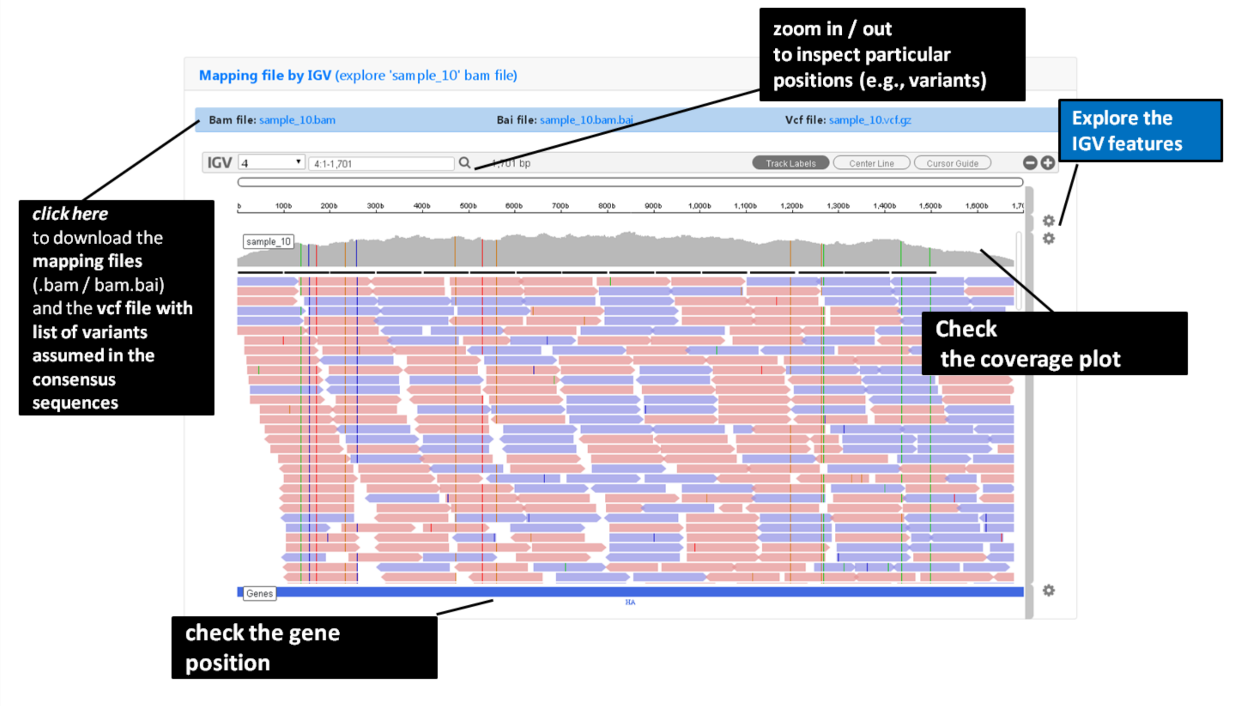

3.5.2 Navigate through global *Projects* outputs

Explore the *Projects menu ("See results" icon)*

The *Projects* tab lists all your projects.

Click on **"See results"** to explore outputs of a given project

The projects outputs are organized by dynamic 'expand-and-collapse' panels containing project-specific outputs (see how to explore each one below). At the bottom of these panels you can explore sample-specific outputs and download the current list of samples.

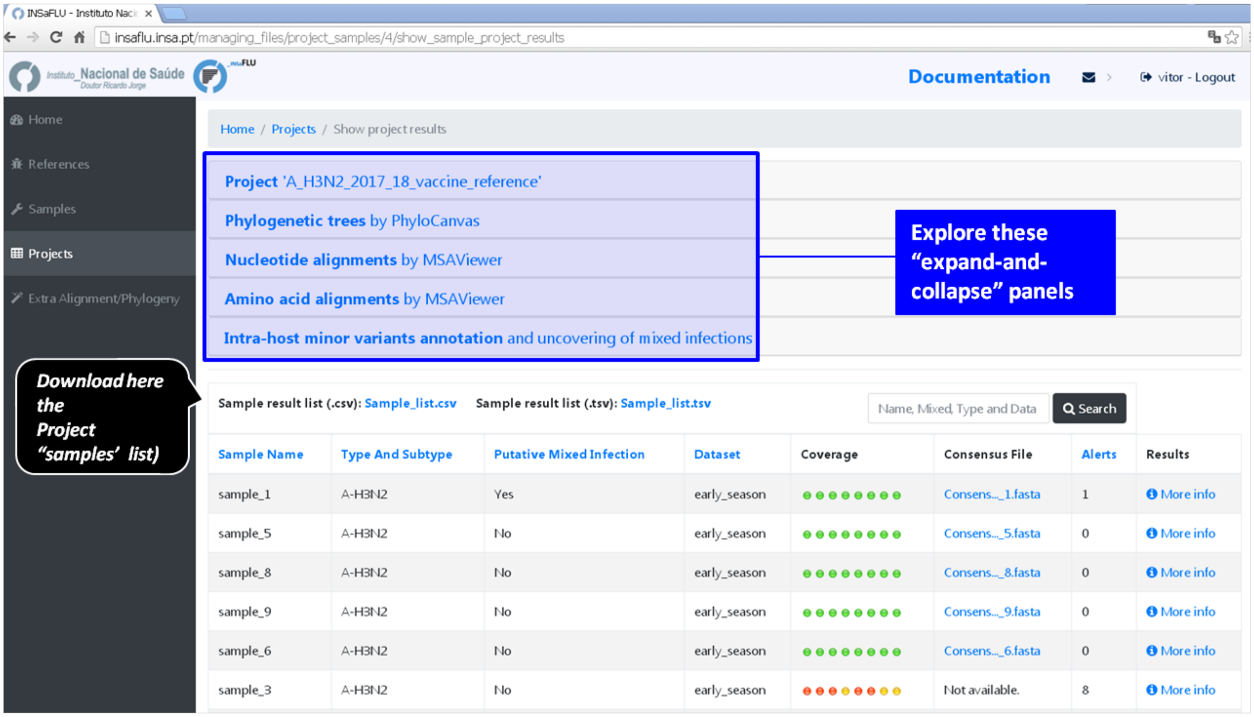

Note: The project samples' list ("Sample_list" file) is automatically re-build and cumulatively updated as more samples are added to the project. This file compiles all samples' metadata as well as sample-specific additional data provided by INSaFLU ("type and subtype/lineage" and "putative mixed infection" data)

The "Sample_list" file can be uploaded, together with associated alignment or phylogenetic data, to visualization tools (see more details on the tab **Uploading data / Uploading Sample metadata and NGS data**)

A. Click on the panel Project 'Project_name' to get an overview on the project

Within this panel you can get an overview on the project (e.g., number of samples processed, reference used, etc), and download project-specific outputs:

- Global **Coverage report**
- **List of all validated variants (SNPs and indels)**
- **List of all minor intra-host single nucleotide variants (iSNVs)**

Note: These tables are automatically re-build and cumulatively updated as more samples are added to the project.

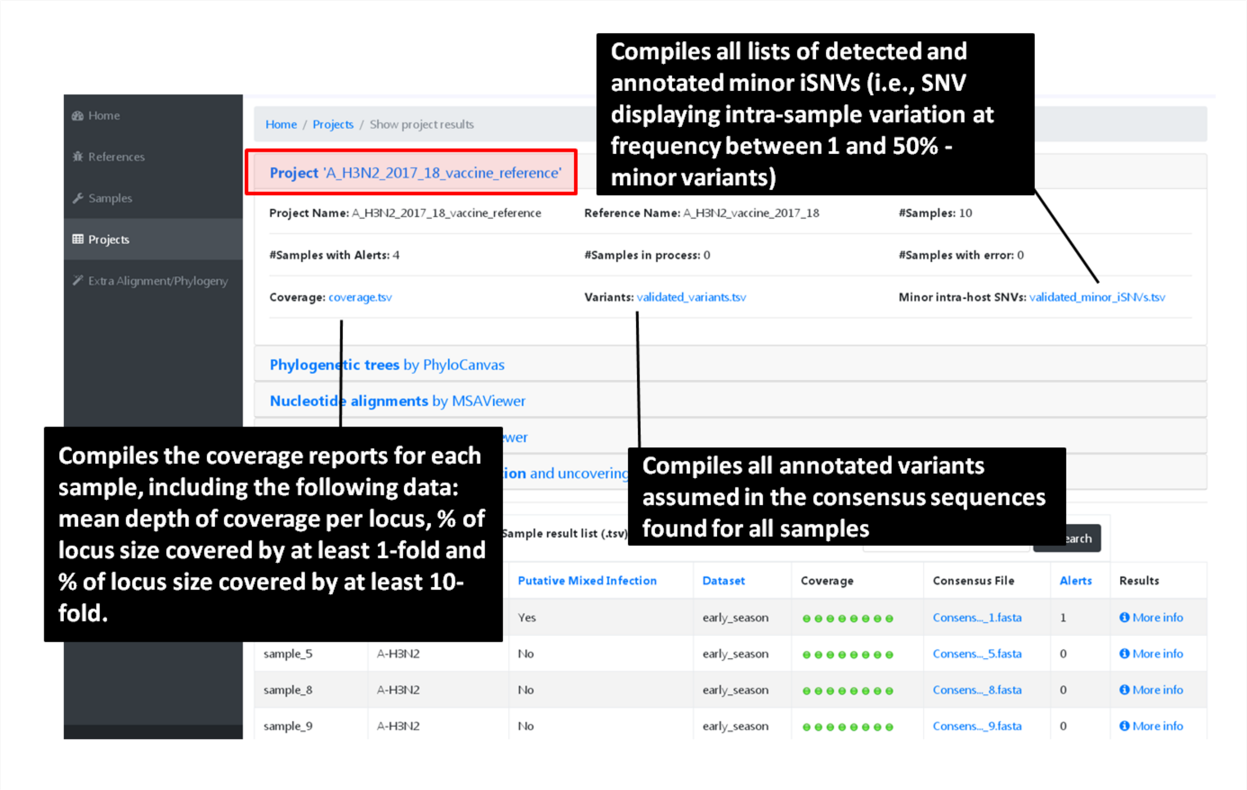

> **Warning:** Validated variants falling within loci not fully covered with 10-fold (color-coded as yellow or red) are still included in the "validated_variants" list (these cases are labeled in the column "VARIANTS in INCOMPLETE LOCUS" as YES), so that users can still retrieve valuable and reliable data (e.g., specific epitope and antiviral drug resistance mutations) from samples with borderline coverage.

B. Navigate through Phylogenetic trees by Phylocanvas

Within this panel you can explore the "whole-genome"-based ("All") and locus-specific phylogenetic trees

Note: Phylogenetic trees are automatically re-build and cumulatively updated as more samples are added to the project.

The Reference virus is included in each phylogenetic tree by default.

Trees are only built when projects have more than one sample.

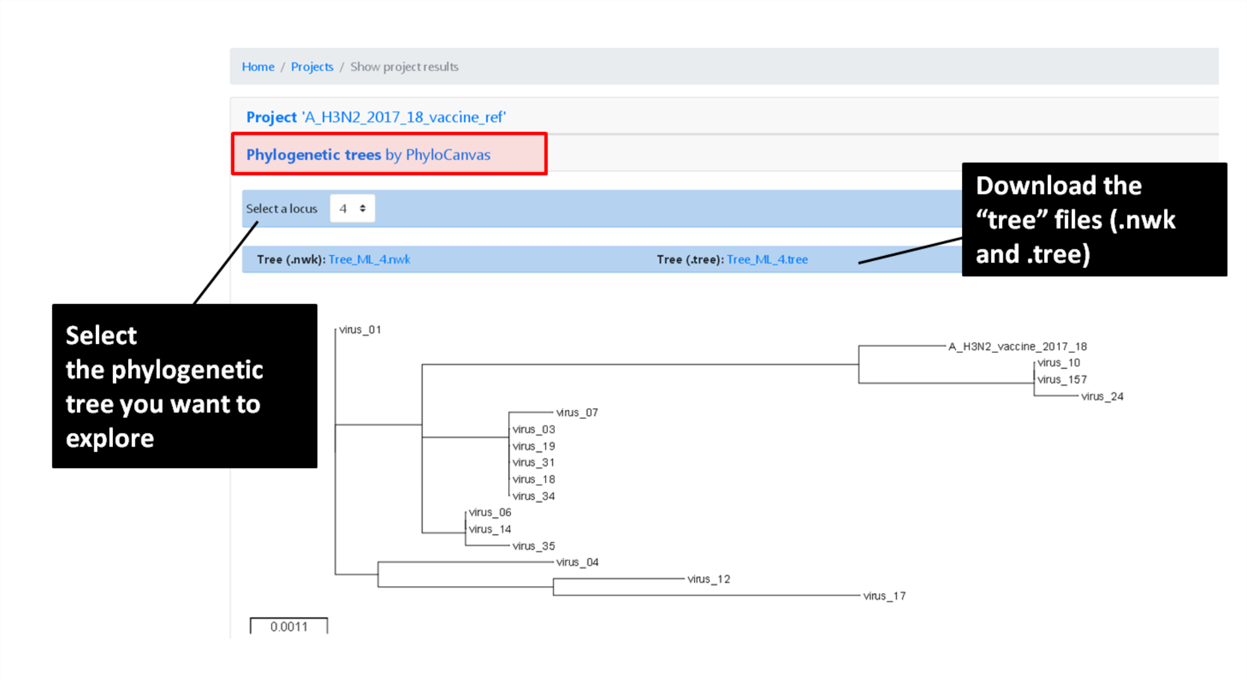

Warning:

- Each locus-specific tree exclusively enrolls samples displaying 100% of that locus covered by 10-fold (color-coded as green in the coverage interactive report).
- The genome-based phylogenetic tree ("All") exclusively enrolls samples displaying all loci all loci with 100% of its length covered by 10-fold (i.e., samples color-coded as green in the coverage interactive report for the all loci panel)

C. Navigate through Nucleotide alignments by MSAViewer

Within this panel you can explore the "whole-genome"-based ("All") and locus-specific nucleotide alignments

Note: Nucleotide alignments are automatically re-build and cumulatively updated as more samples are added to the project.

The Reference sequence is included in each alignment.

Alignments are only built when projects have more than one sample.

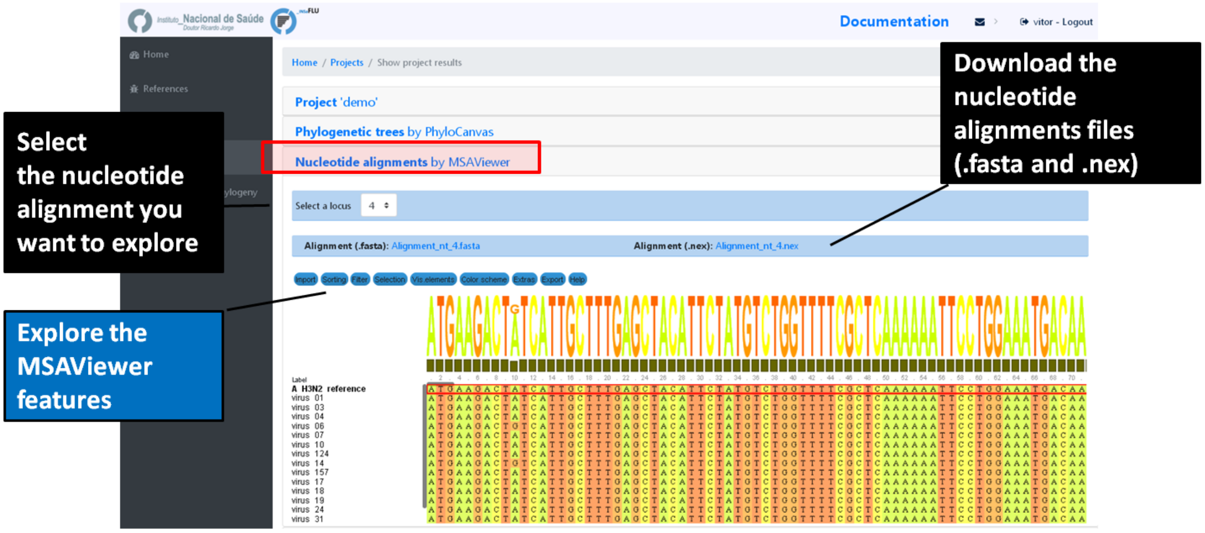

Warning:

- Each locus-specific alignment exclusively enrolls samples displaying 100% of that locus covered by 10-fold (color-coded as green in the coverage interactive report).
- The genome-based nucleotide alignment ("All") exclusively enrolls samples displaying all loci all loci with 100% of its length covered by 10-fold (i.e., samples color-coded as green in the coverage interactive report for the all loci panel)

D. Navigate through Amino acid alignments by MSAViewer

Within this panel you can explore the amino acid alignments for the influenza protein

Note: Amino acid alignments are automatically re-build and cumulatively updated as more samples are added to the project.

The Reference sequence is included in each alignment.

Alignments are only built when projects have more than one sample.

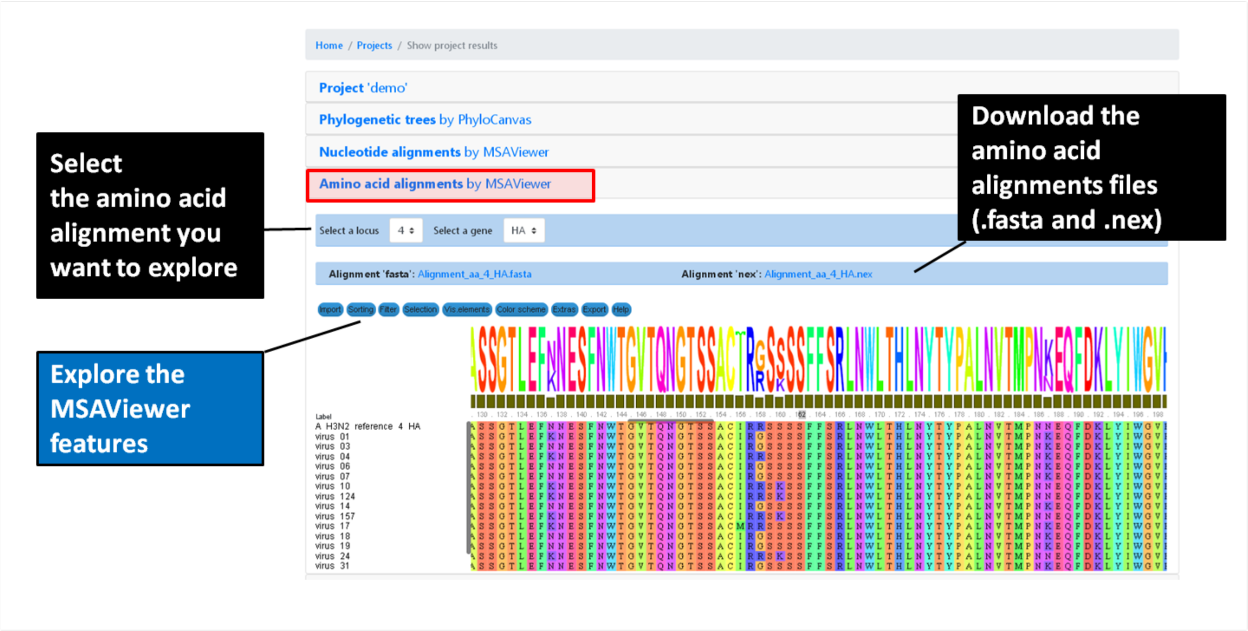

Warning:

- Each amino acid alignment exclusively enrolls samples displaying 100% of that locus covered by 10-fold (color-coded as green in the coverage interactive report).

E. Explore the Intra-host minor variants annotation (and uncovering of putative mixed infections) panel

Within this panel you can explore a graph plotting the proportion of iSNV at frequency at 1-50% (minor iSNVs) and at frequency 50-90%, and download the list of all detected and annotated minor iSNVs (i.e., SNV displaying intra-sample variation at frequency between 1 and 50% - minor variants) for the project.

Note: Both the graph and the list of validated minor iSNVs are automatically re-build and cumulatively updated as more samples are added to the project

You may inspect this plot to uncover infections with influenza viruses presenting clearly distinct genetic backgrounds (so called **'mixed infections'**). A cumulative high proportion of iSNVs at both frequency' ranges is mostly likely to represent a mixed infection, in a sense that the natural intra-patient influenza diversification (that NGS is capable of detecting) is expected to be very low (no more than a few tenths of variants, most of them at frequency <10%)

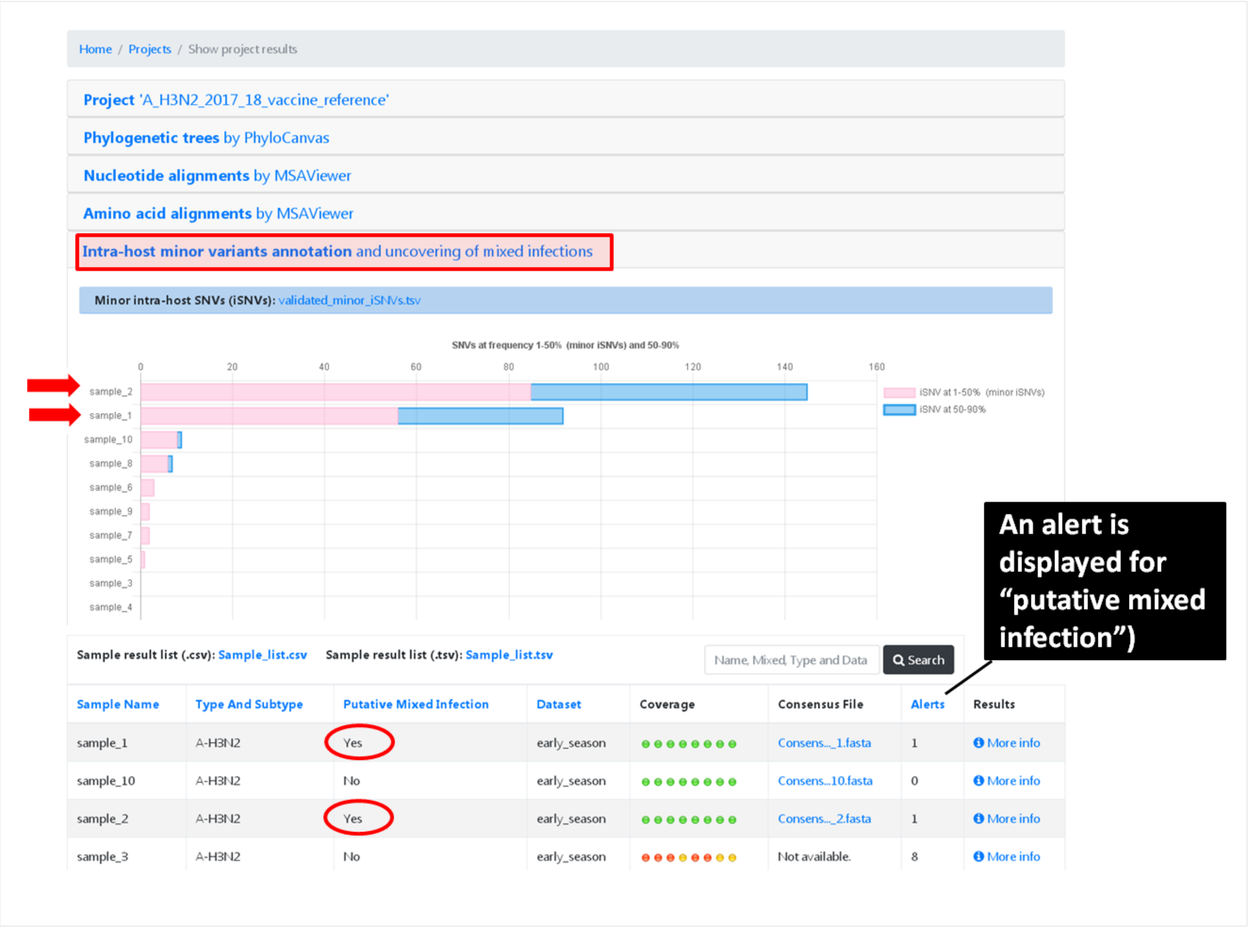

Important:

- INSaFLU flags samples as 'putative mixed infections' based on intra-host SNVs if the following cumulative criteria are fulfilled: the ratio of the number of iSNVs at frequency 1-50% (minor iSNVs) and 50-90% falls within the range 0,5-2,0 and the sum of the number of these two categories of iSNVs exceeds 20. Alternatively, to account for mixed infections involving extremely different viruses (e.g., A/H3N2 and A/H1N1), the flag is also displayed when the sum of the two categories of iSNVs exceeds 100, regardless of the first criterion.
- Note that samples can also be flagged as "putative mixed infections" if if more than one type, HA or NA subtype or lineage is detected (see "Type and subtype identification" module).

Warning:

- By default, samples flagged as "putative mixed infections" are depicted in both alignments and phylogenetic trees. Users are encouraged to inspect the flagged samples by exploring their mapping files (.bam files), "coverage" plots per locus and also the lists of variants.

3.6 Change log

This tab includes a list (chronologically ordered) of notable changes in INSaFLU.

3.6.1 2018

May 14, 2018

- The INSaFLU list of genetic markers "influenza_assign_segments2contigs" was upgraded (now includes 416 sequences), so, from now one, INSaFLU can assign additional close references sequences to your viruses, such as representative virus of distinct genetic sub-groups or seasonal A(H3N2) viruses or representative A(H5N1) sequences of distinct H5 genetic clades.

Latest database can be downloaded here: `INSaFLU_current_genetic_markers_05_06_2018.xlsx`

(the database before this update is also available here: `INSaFLU_genetic_markers_before_14_05_2018.xlsx`

April 9, 2018

- Maximum size per fastq.gz file was upgraded from 50 MB to 300 MB.
  - IMPORTANT NOTE: Files between 50 - 300 MB will be downsized to ~50 MB before analysis by randomly sampling reads using fastq-sample from fastq-tools package https://github.com/dcjones/fastq-tools (developed by Daniel C. Jones dcjones@cs.washington.edu)
- The draft assembly provided by INSaFLU (FASTA format) now additionally includes potential non-influenza specific contigs (i.e., contigs not assigned to any influenza segment / reference by INSaFLU). This feature allows users to better inspect the draft assemblies and reinforces the applicability of INSaFLU for other viruses.

March 9, 2018

- INSaFLU now provides a draft genome assembly (FASTA format) including influenza-specific NODES/contigs. These are identified by screening the SPAdes-derived draft assemblies against an in house database using ABRIcate, which allows assigning NODES/contigs to the corresponding viral segments and to a related reference influenza virus (output: table in ".tsv" format). Please check these new outputs and guide to interpret them at the INSaFLU tab "Samples" / "Extra info" / "Type and subtype/lineage identification". Please also check software settings and parameters at the "Data analysis" tab of this Documentation.

  This new feature reinforces the application of INSaFLU to:

  - analyse viruses for which a close related whole-genome sequence is not available (e.g., avian influenza) at the INSaFLU or other databses (NCBI, GISAID, etc);
  - investigate reassortments
  - disclose mixed infections

January 25, 2018

- INSaFLU 1.0.0 is released for the scientific community at https://insaflu.insa.pt

  INSaFLU ("INSide the FLU") is an bioinformatics free web-based suite that deals with primary NGS data (reads) towards the automatic generation of the output data that are actually the core first-line "genetic requests" for effective and timely influenza laboratory surveillance. While INSaFLU has indeed some influenza-specific features (e.g., automatic type/subtype identification), there is no restrictions to use it for other viruses.

  Main highlights:

  - open to all, free of charge, user-restricted accounts

- applicable to NGS data collected from any amplicon-based schema
- allows advanced, multi-step software intensive analyses in a user-friendly manner without previous training in bioinformatics
- automatic identification of influenza type and subtype/lineage, detection of putative mixed infections and intra-host minor variants
- allows integrating data in a cumulative manner, thus fitting the analytical dynamics underlying the continuous epidemiological surveillance during flu epidemics
- outputs are provided in nomenclature-stable and standardized formats and can be explored in situ or through multiple compatible downstream applications for fine-tune data analysis and visualization
