# Supplementary material for: INSaFLU: an automated open web-based bioinformatics suite “from-reads” for influenza whole-genome-sequencing-based surveillance
Source: Genome Med. 2018 Jun 29;10:46. doi: 10.1186/s13073-018-0555-0 (PMC6027769; doi:10.1186/s13073-018-0555-0)
Supplement: Supplementary file 3 — Figure S1. INSaFLU graphical output plotting the number of iSNVs at frequencies 1–50% (minor iSNVs) and 50–90% obtained for dataset 1. Figure S2. INSaFLU testing with artificial mixtures of A(H3N2) viruses. A. INSaFLU graphical output plotting the number of iSNVs at frequencies 1–50% (minor iSNVs) and 50–90%. B. Phylogenetic tree based on “whole-genome” consensus sequences obtained for dataset 3. (PDF 977 kb) [file 13073_2018_555_MOESM3_ESM.pdf]

Additional file 3A

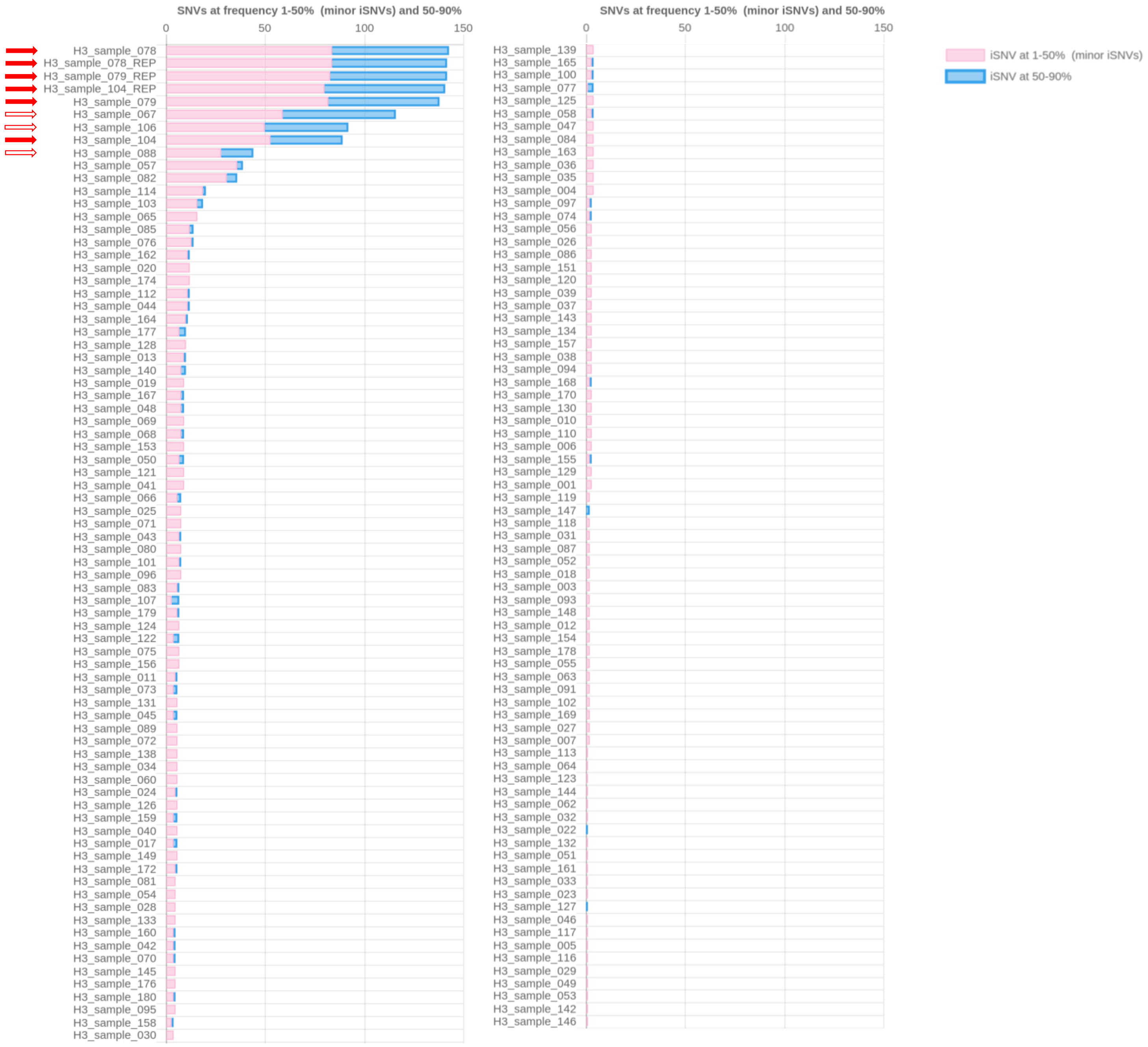

Figure S1. INSaFLU graphical output plotting the number of iSNVs at frequencies 1-50% (minor iSNVs) and 50-90% obtained for dataset 1. Dataset 1 enrolls 192 samples from A(H3N2) viruses that were used for INSaFLU testing and validation. Sample replicates (i.e., processed in independent PCR and NGS runs) were also included in this dataset and are labeled with a "REP" suffixes. This dataset 1 was particularly useful for the empirical definition of criteria for detection of "putative mixed infections" based on of the ratio of number of iSNVs falling into the two indicated categories, as it includes a few "true" mixed infections (labeled with filled red arrows) confirmed upon independent PCR and NGS. INSaFLU flags samples as "putative mixed infections" based on the number of iSNVs at frequency 1-50% ("A") and 50-90% ("B") when: *i*) $0.5 < A/B < 2.0$ and $A+B > 20$; and/or, *ii*) $A+B > 100$. As expected, with exception of samples flagged as "putative mixed infections" according to INSaFLU criteria (all samples flagged with red arrows), samples revealed just a few iSNVs, most of them at low frequency (1-10%). The robustness of the defined criteria was also consolidated with artificial mixtures of dataset 3 (see Additional file 4). For figure simplification, samples revealing no iSNVs are not shown. Still, the full graph (including source data), as well as all other derived outputs from this dataset, can be explored at INSaFLU webpage through the open "demo" account.

Additional file 3B

INSaFLU testing with artificial mixtures of A(H3N2) viruses. This test refers to the INSaFLU' analysis of a dataset (dataset 3) enrolling artificial mixtures (in triplicate) of A(H3N2) viruses at various proportions previously generated by Shepard and colleagues [17] (available at https://wonder.cdc.gov/amd/flu/irma/). Upon reads's quality analysis and improvement, samples were run in an INSaFLU project using the whole-genome consensus sequence obtained for the "PCR1_100_0" sample as the reference sequence. Samples' names in Figures S2A and S2B indicate both the triplicate number (PCR1, 2 or 3) and the different relative proportions of the two mixed same-subtype viruses.

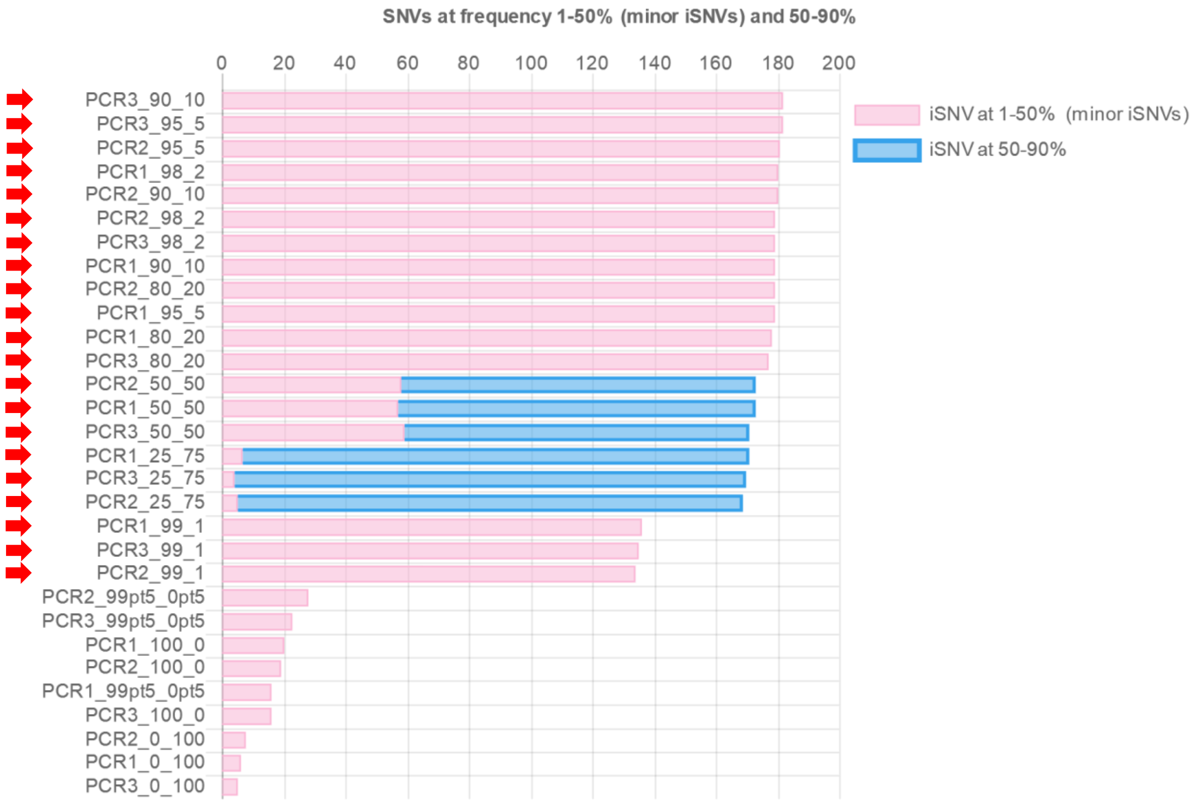

Figure S2A. INSaFLU graphical output plotting the number of iSNVs at frequencies 1-50% (minor iSNVs) and 50-90% obtained for dataset 3. INSaFLU takes advantage of the ratio of number of iSNVs falling into the two indicated categories to find "putative mixed infections" (see a detailed description of the criteria in the main text). Samples flagged as "putative mixed infections" (labeled with a red arrow) fulfilled the INSaFLU criteria (criterion 1: 0.5 < A/B < 2.0 and A+B > 20; and/or criterion 2: A+B > 100, where "A" = number of iSNVs at frequency 1-50% and "B" = number of iSNVs at frequency 50-90%), meaning that, using this dataset, INSaFLU detected these same sub-type mixed infections at relative frequencies of as far as 99:1.

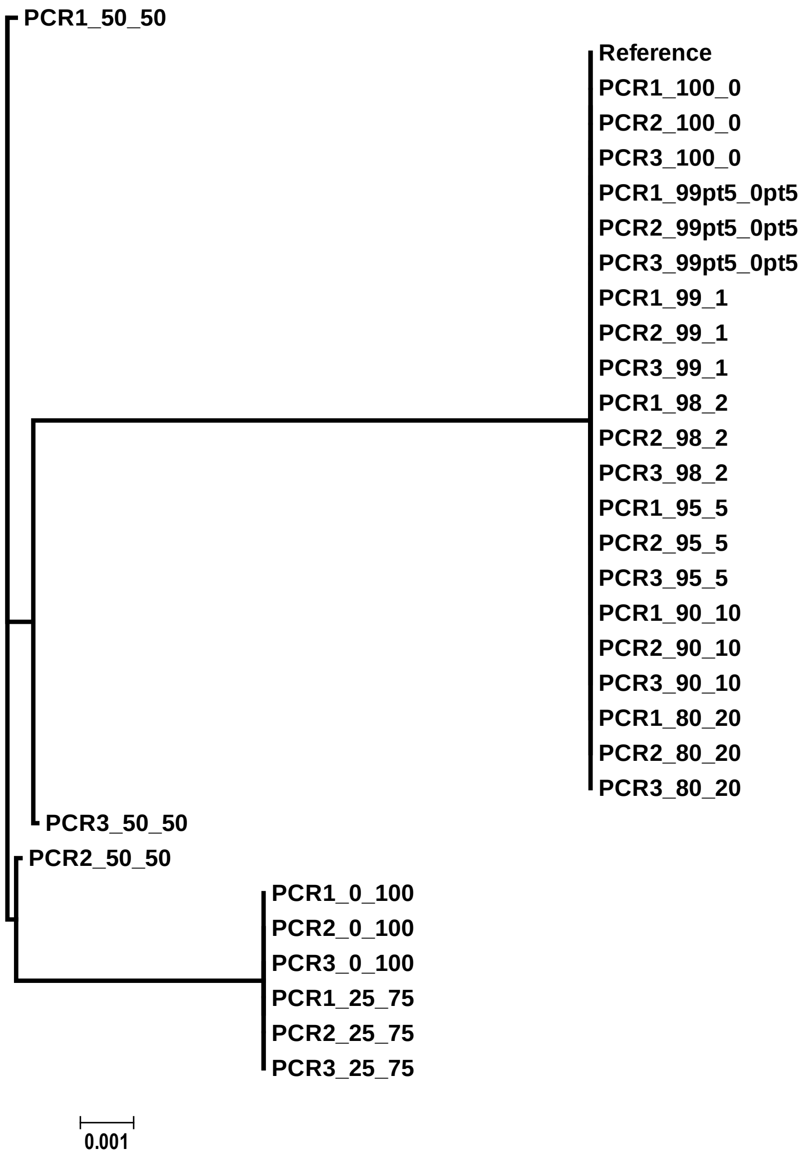

Figure S2B. Phylogenetic tree based on "whole-genome" consensus sequences obtained for dataset 3, showing that INSaFLU yielded match consensus sequences for all sample mixtures (and replicates) sharing the dominant virus. As expected, 50:50 mixtures yielded artificial consensus sequences that do not resemble any of the mixed stocks. The phylogenetic tree was built using FastTree2, as described in "Alignment/phylogeny section". Scale refers to the number of substitutions *per* site (in a total of 13133 nucleotides compared).
